# A novel protein encoded by circCOPA inhibits the malignant phenotype of glioblastoma cells and increases their sensitivity to temozolomide by disrupting the NONO–SFPQ complex

**DOI:** 10.1038/s41419-024-07010-z

**Published:** 2024-08-25

**Authors:** Dazhao Peng, Cheng Wei, Boyuan Jing, Runze Yu, Zhenyu Zhang, Lei Han

**Affiliations:** 1 Tianjin Neurological Institute, Key Laboratory of Post-Neuro injury, Neuro-repair and Regeneration in Central Nervous System, Ministry of Education and Tianjin City, Tianjin Medical University General Hospital, Tianjin, China; 2 Department of Neurosurgery, The First Affiliated Hospital of Zhengzhou University, Zhengzhou, Henan China

**Keywords:** CNS cancer, Non-coding RNAs

## Abstract

Glioblastoma (GBM) represents a primary malignant brain tumor. Temozolomide resistance is a major hurdle in GBM treatment. Proteins encoded by circular RNAs (circRNAs) can modulate the sensitivity of multiple tumor chemotherapies. However, the impact of circRNA-encoded proteins on GBM sensitivity to temozolomide remains unknown. Herein, we discover a circRNA (circCOPA) through the circRNA microarray profile in GBM samples, which can encode a novel 99 amino acid protein (COPA-99aa) through its internal ribosome entry site. Functionally, circCOPA overexpression in GBM cells inhibits cell proliferation, migration, and invasion in vitro and growth in vivo. Rather than itself, circCOPA mainly functions as a suppressive effector by encoding COPA-99aa. Moreover, we reveal that circCOPA is downregulated in GBM tissues and high expression of circCOPA is related to a better prognosis in GBM patients. Mechanistically, a heteromer of SFPQ and NONO is required for double-strand DNA break repair. COPA-99aa disrupts the dimerization of NONO and SFPQ by separately binding with the NONO and SFPQ proteins, thus resulting in the inhibition of proliferation or invasion and the increase of temozolomide-induced DNA damage in GBM cells. Collectively, our data suggest that circCOPA mainly contributes to inhibiting the GBM malignant phenotype through its encoded COPA-99aa and that COPA-99aa increases temozolomide-induced DNA damage by interfering with the dimerization of NONO and SFPQ. Restoring circCOPA or COPA-99aa may increase the sensitivity of patients to temozolomide.

## Introduction

GBM ranks as the most prevalent form of primary malignant brain tumor [1], accounting for approximately 75% of all malignant tumors of the central nervous system [2]. Currently, the treatment of GBM primarily involves surgery combined with radiotherapy, temozolomide (TMZ) chemotherapy, immunotherapy and tumor-treating fields (TTFields) [3–5]. Unfortunately, patients with GBM have a dismal prognosis.

Circular RNAs (circRNAs) are a class of single-stranded noncoding RNAs with covalent closed-loop structures [6]. They are considered as diagnostic markers and therapeutic targets for many types of tumors, including GBM [7, 8]. An increasing number of studies have shown that aberrantly expressed circRNAs play important roles in the occurrence and progression of GBM [7]. For instance, circRNAs can disrupt glycolysis and promote the MDA5-mediated immune response by binding with TKFC proteins, thus suppressing the progression of glioma [9]. By functioning as miRNA sponges, circRNAs can promote glioma tumorigenesis [10]. Importantly, through internal ribosome entry sites (IRESs), N6-methyladenosine modifications or infinite open reading frames, circRNAs can encode unique peptides/proteins. The impact of circRNA-encoded proteins/peptides on the malignant phenotype of GBM has received widespread attention [11, 12]. CircRNAs are increasingly recognized as potential targets for the treatment of various diseases, including GBM [13–15].

TMZ presents the primary chemotherapy drug for GBM treatment and causes cytotoxicity by causing DNA double-strand break (DSB) [16]. The response of DNA damage repair can reverse the damage and lead to TMZ chemotherapeutic resistance [17]. Many studies have reported that the NONO–SFPQ complex is a heterodimer [18, 19] and is involved in DNA damage repair [20–23]. NONO and SFPQ exhibit over 70% sequence similarity in their common core region of around 300 amino acids, known as the DBHS domain, which encompasses a coiled-coil region, a NONO/paraspeckle (NOPS) domain and two RNA recognition motif domains (RRM1 and RRM2) [24, 25]. The RRM1 domain is typically essential for RNA binding. The dimerization of NONO and SFPQ is facilitated by RRM2, NOPS and part of the coiled-coil, which is required for stable protein structure and functional repertoire in vivo [25, 26]. However, whether circRNAs can regulate GBM sensitivity to TMZ by interacting with the NONO–SFPQ complex is unknown.

Through a circRNA microarray profile, we observed a decrease in circCOPA expression levels in GBM tissues compared to paired normal brain tissues. CircCOPA suppressed the proliferation, migration, and invasion of GBM cells. Importantly, it was verified that circCOPA mainly inhibited the malignant phenotype of GBM by encoding the new protein COPA-99aa. COPA-99aa separately bound with NONO and SFPQ to disrupt the dimerization of NONO and SFPQ. This mutual interaction inhibited the proliferation and invasion, and decreased DNA damage repair mediated by the NONO–SFPQ complex and increased TMZ-induced DNA damage. Collectively, our results showed that circCOPA encoded a novel protein, COPA-99aa, which inhibited the GBM malignant phenotype by disrupting the NONO–SFPQ complex.

## Materials and methods

### Human GBM and paired adjacent brain tissues

Four paired GBM tissues and their paired normal brain tissues were collected from the First Affiliated Hospital of Zhengzhou University. These four paired samples were analyzed via circRNA microarray. Meanwhile, 36 pathologically diagnosed primary GBM patient samples were also collected (Supplementary Table 1). The neuropathologist verified the pathological characteristic of each tissue sample. This study was performed according to the principles of the Declaration of Helsinki. This study was approved by the Human Scientific Ethics Committee of the First Affiliated Hospital of Zhengzhou University (No. 2019-KY-176) and the informed consent was written and provided by patients after being fully informed.

### Cell culture

The 293 T, UC2 astrocyte, GBM cells (including U87, U251, LN18, SNB19, and LN229 cell) were stably cultured for a long time by our group. Mycoplasma testing and STR sequencing were implemented. The human immortalized astrocyte cell line UC2 was kindly provided by Prof. Shizhu Yu and Xuexia Zhou [27]. All cell lines were cultured in Dulbecco’s modified Eagle’s medium containing 10% fetal bovine serum (FBS, BI serum, Israel). They were cultured in an incubator (37 °C, 5% CO_2_).

### CircRNAs microarray hybridization and data analysis

The circRNAs microarray was performed by KangChen Bio-tech (Shanghai, China). The “fold change” between the groups for each circRNA was used to compute the circRNA differences between the GBM and paired adjacent normal brain tissues. The statistical significance of the difference was estimated by t-test. The differentially expressed circRNAs (|fold changes| ≥ 1.3 and *p*-values ≤ 0.05) were subjected to statistical analysis of type and chromosome distribution.

Using the OmicShare Tools (https://www.omicshare.com/tools), Volcano Plot filtering identified the differentially expressed circRNAs with statistical significance between two groups. Hierarchical Clustering identified the distinguishable circRNAs expression pattern among samples.

### Bioinformatics analysis

GSE92322 and GSE165926 datasets in Gene Expression Omnibus (GEO) database were download [28]. Differentially expressed circRNAs were analyzed and screened by the GEO2R tool. The encoding ability of circRNAs was analyzed in circbank (http://www.circbank.cn/index.html) [29], circRNADb (http://reprod.njmu. edu.cn/cgi-bin/circrnadb/circRNADb.php) [30], and TransCirc (https://www.biosino. org/transcirc/) [31] online databases. Additionally, we obtained the IRES sequence of circRNAs in circRNADb database. Through the RNAfold WebServer (http://rna.tbi.univie.ac.at/cgi-bin/RNAWebSuite/RNAfold.cgi), the secondary structures of IRES sequences were obtained and the minimum free energy structure was download to further study.

### Plasmid, siRNA, and lentiviral

CircCOPA-overexpressing plasmid was constructed by using GV728 vector (Genechem, Shanghai, China). COPA-99aa-3×Flag cDNA was cloned into the GV104 vector (provided by Prof. Jinjin Sun) through the KpnI and PacI sites. CircCOPA-3×Flag and circCOPA-3×Flag-Del plasmids were synthesized employing the pCD-5 ciR vector (Geneseed, Guangzhou, China) as the backbone. The plasmids were transfected into cells using Lipofectamine 3000 (L3000015, Thermo Fisher Scientific, USA).

The short interfering RNA (siRNA) of circCOPA was synthesized by GenePharma (Shanghai, China). Lipofectamine RNAiMax (Thermo Fisher Scientific, USA) was used to transfected siRNA to cells. The lentivirus carrying artificial circCOPA expression plasmid was further constructed (Genechem, Shanghai, China). Then, the stable U87 cell line was generated by transfecting with this lentivirus, followed by selection with 2 μg/ml puromycin for 72 h.

### Dual-luciferase assay

Dual-luciferase reporter gene was generated through chemical gene synthesis (GENEWIZ, Suzhou, China). Using the psicheck2 vector, the IRES sequences of hsa_circ_0008661, circSLC25A24 and circYWHAZ as well as truncated IRES sequences of circCOPA were cloned between renillaluciferase (Rluc) and luciferase (Luc) by NotI and MluI restriction enzyme sites. The negative control plasmid lacked the IRES sequence. Using the dual-luciferase reporter assay system (E1910, Promega, USA), we measured the relative luciferase activity (Luc/Rluc). The relative luciferase activity was examined through a microplate reader (Synergy2, BioTek, USA).

### Reverse transcription and real-time PCR (RT-qPCR)

Total RNA of cell lines was extracted using TRIzol reagent (9109, TaKaRa, Japan). According to the manufacturer’s instructions, reverse transcription for mRNA and circRNAs was performed with GoScript Reverse Transcription system (A5001, Promega, USA). Polymerase chain reaction was subsequently performed with reverse-transcribed cDNA. A 2% agarose gel was used to test the PCR product. RT-qPCR was performed on ABI QuantStudio 3 using 2×SYBR Green qPCR Master Mix (Low ROX) (B21702, Selleck, USA). The PCR system was 20 μL (10 μM forward and reverse primers: 2 μL, 2×SYBR Green: 10 μL, cDNA: 2 μL, RNase free water: 6 μL). The relative expression levels of mRNA and circRNAs were calculated according to 2^−ΔΔCT^. The relative expression was normalized to the GAPDH mRNA in each sample. The primer sequences for each gene are summarized in (Supplementary Table 2).

### Actinomycin D assay

The actinomycin D (HY-17559, MedChemExpress, USA) was used to treat 293T cells (2 μg/ml). The total RNA of cells was obtained after 0, 4, 8, and 12 h of actinomycin D treatment. The expression of circCOPA and COPA mRNA was analyzed through RT-qPCR. The values of the 0 h group were normalized and the relative expression levels of circCOPA and COPA mRNA were calculated.

### RNase R treatments

We used the RNase R (RNR07250, Lucigen, USA) to treat the total RNA (37 °C, 15 min). The resistance of circCOPA to RNase R digestion was validated through RT-qPCR.

### Isolation of nuclear and cytoplasmic RNA

The subcellular localization of circRNA was verified with reference to the method of Wei et al. [32]. Briefly, 4 × 10^6^ cells were collected. We used nuclear and cytoplasmic extract to detect the expression of circCOPA, COPA mRNA, b-actin, U6 and GAPDH.

### RNA fluorescence in situ hybridization (FISH)

We first synthesized the Cy3-labeled oligonucleotide probes of circCOPA (GenePharma Jiangsu, China). Poly-L-ornithine (Sigma-Aldrich) was used to pretreat coverslips for 24 h followed by implantation of cells onto them. An RNA FISH kit (GenePharma) was used to performed FISH. The nuclei were stained (4,6-diamidino-2-phenylindole). The sequence of circCOPA probe is: 5′-TTAACACACATTCATGCGCCAGATC-3′.

### The proliferation, migration and invasion of GBM cells

CCK8 and EdU assays were used to detect the cell proliferation.CCK8 reagent was purchased from APExBIO (K1018, USA). The cells (1.0 × 10^4^ cells/well) were seeded into 96-well plates. The test was lasted for 5 consecutive days. EdU assay Kit (Cell-Light EDU Apollo488 In Vitro Kit) was obtained Ribobio Co., Ltd (Guangzhou, China). According to the kit instructions, the experiment was strictly performed.

Wound healing assay examined the ability of cell migration. Cells (2 × 10^5^ cells/well) were seeded into the 6-well plate and cultured 24 h. The images were collected at 0, 12, and 24 h after the corresponding treatment (IX81, Olympus Company, Japan). Using ImageJ software, we calculated and analyzed the cell migration rate.

Transwell assay was used to detect the ability of cell migration and invasion. The chamber coated with Matrigel matrix glue (Corning, USA) tested the ability of cell invasion. The images of cell staining were collected (BX53, Olympus Company, Japan). Using ImageJ software, we counted the number of cells.

### Western blotting (WB)

Using RIPA lysis buffer (R0020, Solarbio, China) with protease and phosphatase inhibitor cocktail (78441, Thermo Fisher Scientific, USA), proteins were extracted. BCA kit (PC0020, Solarbio, China) was used to quantified proteins. SDS-PAGE gels (8%-12%) were used to separate total proteins. Then, they were transferred by using PVDF membranes (Millipore, USA). After blocking, the membranes were incubated with corresponding primary antibodies. Finally, the membranes were incubated with secondary antibodies (Invitrogen). The chemiluminescence signal was detected through chemiluminescence system (Bio-Rad, Hercules, EDA USA). The primary antibodies included: Flag (F1804, Sigma-Aldrich), γH2AX (ab81299, abcam, USA), SFPQ (sc-374502, Santa Cruz Biotechnology) and NONO (11058-1-AP, proteintech).

### Co-Immunoprecipitation (co-IP)

The Pierce™ IP lysis buffer (87788, Thermo Fisher Scientific, USA) with protease and phosphatase inhibitor cocktail (78441, Thermo Fisher Scientific, USA) was used to lyse cells. The lysates were collected and cleared via incubation with protein A/G PLUS agarose (sc-2003, Santa Cruz Biotechnology, USA) (30 μl, 4 °C, 1 h). The pre-cleared supernatant was subjected to immunoprecipitation using the primary antibodies and 60 μl of protein A/G gel at 4 °C overnight. Finally, we collected all protein complexes, which were analyzed by performing WB. The antibodies were following: NONO (sc-376865, Santa Cruz Biotechnology), SFPQ (15585-1-AP, proteintech), Flag (F1804, Sigma-Aldrich) and IgG (30000-0-AP, proteintech) for immunoprecipitation.

### LC–MS/MS analysis

Total protein was separated via SDS–PAGE (10%). The SDS–PAGE gel was performed Coomassie blue staining. The band was manually excised and then send to Shanghai Applied Protein Technology Co. Ltd (Shanghai, China) to further analyzed. The QExactive mass spectrometer (Thermo Fisher Scientific, Waltham, MA, USA) was used to analyze the digested peptides. Through the National Center for Biotechnology Information nonredundant protein database with Mascot (Matrix Science, Boston, MA, USA), fragment spectra were analyzed.

### RNA sequencing (RNA-seq)

U87 cells was transfected with COPA-99aa-3×Flag or control plasmid. RNA sequencing was performed by Gene Denovo Biotechnology Co. (Guangzhou, China). We identified the differentially expressed genes (DEGs, |fold change| > 2 and FDR < 0.05). Kyoto Encyclopedia of Genes and Genomes (KEGG), Gene Ontology (GO) and Disease Ontology (DO) enrichment analysis was performed for DEGs.

### Molecular docking

Using I-TASSER (Iterative Threading ASSEmbly Refinement) serve (https://zhanggroup.org/I-TASSER/), the 3D structure of COPA-99aa was predicted. The 3D structure of the NONO–SFPQ complex was obtained from RCSB Protein Data Bank (PDB ID: 6WMZ). Molecular docking simulations of COPA-99aa and NONO–SFPQ were conducted by using ClusPro2.0 (https://cluspro.org). Results were generated and modified via PyMOL.

### Immunofluorescence

4% formaldehyde (Fisher) was used to fix cells for 10 min. Cells were permeabilized with 0.1% Triton X-100 and blocked with 1% BSA in PBS. Then, immunostaining was performed using the γH2AX primary antibody (ab81299, abcam, USA), Flag (F1804, Sigma-Aldrich) and Alexa Fluor™ 594 secondary antibody (A12381, Thermo Fisher Scientific, USA). DAPI (P36971, Thermo Fisher Scientific, USA) was incubated in the dark. Finally, the fluorescence image was collected.

### Comet assays

Comet assays were performed according to manufacturer’s protocol (C2041M, Beyotime, China). Briefly, cells were trypsinized, washed with cold PBS, collected by centrifugation. The cell density was 1 × 10^6^ cells/ml by adding an appropriate amount of PBS. The first layer of 1% normal melting agarose gel was prepared on a slide and left at room temperature overnight. Mix 10^4^ cells with 75 µl of 0.7% low melting agarose, then quickly add them on the first layer of gel, gently spread them out with a pipette tip to cover the entire first layer of gel, left for 10 min at 4 °C. Lysis solution (Lysis Buffer: DMSO = 9:1 v/v ratio) was prepared and place it at 4 °C for pre-cooling. Place the slide in a horizontal bath, pour in the electrophoresis buffer and leave it at room temperature for 20–60 min. Electrophoresis was performed in a horizontal bath (~0.75-1 V/cm, 20 min) under light and ice bath conditions. After electrophoresis, the slide was placed in a plate, neutral buffer was added, and neutralized 1–3 times at 4 °C for 5–10 min each time. Add 20 µl Propidium Iodide Solution to the slide and stain away from light for 10-20 min. It was then washed with ultrapure water three times, covered with a cover glass, and viewed under a fluorescence microscope. All images were further analyzed using Open Comet software and Tail Moment was selected as an indicator to assess the extent of DNA damage.

### Intracranial tumor model construction

Animal experiments are conducted under the guidance approved by Tianjin Medical University Animal Care and Use Committee. The four-week-old BALB/c female nude mice was obtained (Beijing HFK Bioscience Co., LTD, China). U87 cells (5 × 10^5^) were co-infected with GFP-luciferase lentiviruses (GenePharma, China) and circCOPA expressing lentiviruses (Genechem, Shanghai, China). U87 cells (5 × 10^5^) solely infected with GFP-luciferase lentiviruses were used as a control. Using microinfusion pump and stereotactic instrument (Stoelting Co., USA), cells were implemented into the intracranial striatum of nude mice. U87 cells (5 × 10^5^) infected only with GFP-luciferase lentivirus were used as control group to construct intracranial tumor model. After 10 min intraperitoneal injection of D-fluorescein (150 mg/kg, E1605, Promega, USA), bioluminescent imaging was captured using the IVIS imaging system (PerkinElmer, USA). All mice were monitored every 7 days and tumor growth status in different groups was monitored by bioluminescent images. Mice were fed until they displayed neurological symptoms or until reaching 50 days.

### Statistical analysis

The statistical calculation and image were produced by GraphPad Prism 8. Unpaired *t*-test was used to compare the difference between the two groups. The difference between multiple groups was analyzed by one-way ANOVA. The log-rank test was use to examine the survival analysis. Error bars represent three independent experiments, *p < 0.05, **p < 0.01, ***p < 0.001, ****p < 0.0001. No significant = ns and *p* < 0.05 was regarded as statistically significant.

## Results

### Screening circRNAs with potential encoding ability

**Fig. 1:**
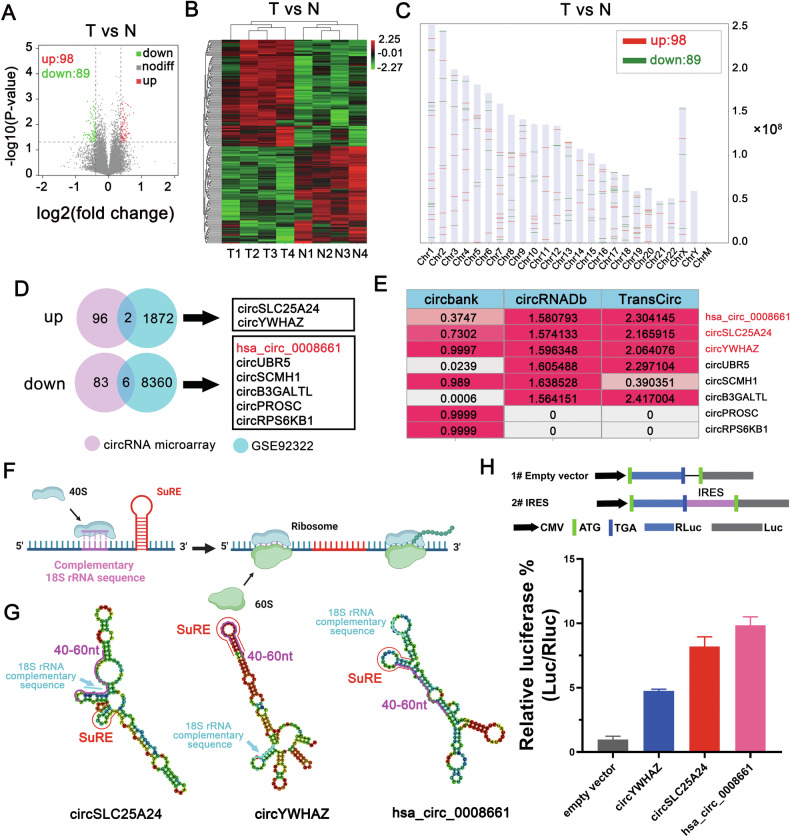
Screening of circRNAs with encoding ability. **A** Four paired GBM and paired normal brain tissues were subjected to microarray analysis. A volcano plot revealed 187 differentially expressed circRNAs (*p* < 0.05 and |fold change| > 1.3). Among these circRNAs, 98 were upregulated and 89 were downregulated in GBM. *N* = adjacent brain tissue samples, T = GBM tissue samples. **B** Heatmap showing that the differentially expressed circRNAs between GBM tissues and adjacent brain tissues were clearly distinguishable. **C** The chromosomal distribution of differentially expressed circRNAs. **D** A total of 8 common differentially expressed circRNA candidates were identified in our microarray data and GSE92322 dataset. Among these 8 candidates, 2 circRNAs were upregulated and 6 circRNAs were downregulated in both datasets. **E** Evaluation of the potential encoding ability of 8 circRNAs through the circBank, circRNADb and TransCirc online databases. The “coding_prob” score in circBank, “R Score” in circRNADb and “evidence score” in TransCirc were used as indicators to evaluate the coding potential of circRNAs. **F** An illustration of the 18S rRNA complementary sequence and SuRE element facilitating circRNA translation. The SuRE causes a pause for RNA unwinding, increasing the chance for the 18S rRNA complementary sequence to bind to the 18S rRNA on the ribosome to facilitate circRNA translation. **G** Potential IRES sequences of circSLC25A24, circYWHAZ and hsa_circ_0008661 and their secondary structure features were predicted by the RNAfold WebServer. **H** The IRES activity was examined. Upper panel, IRES sequences in circSLC25A24, circYWHAZ and hsa_circ_0008661 were cloned from Rluc and Luc reporter genes with independent start and stop codons. In the lower panel, a luciferase assay was used to examine the relative luciferase activity of Luc/Rluc (*n* = 3). Data represent means ± SD of three independent experiments (bar plots).

To perform circRNA profiling, we generated a circRNA microarray of RNase R-digested total RNA from four GBM specimens and paired normal brain specimens. It was reported that 13,617 circRNAs were differentially expressed between GBM and adjacent brain tissues. Among these differentially expressed circRNAs, 98 circRNAs were upregulated in GBM, while 89 circRNAs were downregulated in GBM (Fig. 1A, B). The chromosomal distribution of these differentially expressed circRNAs was analyzed (Fig. 1C). The results showed that these circRNAs were distributed on all chromosomes except the Y chromosome. The upregulated circRNAs were mainly located on chromosome 4 and chromosome 7, and the downregulated circRNAs were mainly distributed on chromosome 17 and chromosome 18. Among them, circRNAs derived from chromosome 11 were highly expressed in GBM tissues. Next, a microarray dataset (GSE92322) comprising 5 primary GBM and 5 adjacent brain specimens from the GEO database was cross-matched with our microarray data. Eight common differentially expressed circRNAs were identified in both datasets (Fig. 1D). Among these 8 candidate circRNAs, 2 circRNAs were upregulated, and 6 circRNAs were downregulated in both datasets.

Recently, the potential encoding ability of circRNAs has been frequently reported [11, 33]. Thus, we first evaluated the potential encoding ability of the above 8 candidate circRNAs in the circBank, circRNADb and TransCirc databases. As shown in Fig. 1E, three candidate circRNAs (hsa_circ_0008661, circSLC25A24, and circYWHAZ) showed potential coding ability in all three databases. Chen et al. reported that the 18S rRNA complementary sequence and the SuRE element (near the 40–60 nt position from the first nucleotide of the IRES) on circRNA IRES play crucial roles in promoting the initiation of translation for endogenous circRNAs [34]. Specifically, the SuRE element may pause RNA unwinding, increasing the interaction between the 18S rRNA on the ribosome and the 18S rRNA complementary sequence on the IRES to promote circRNA translation (Fig. 1F). Thus, we obtained potential IRES sequences of the above three circRNAs from the circRNADb database and further analyzed their secondary structural features through the RNAfold WebServer. The results showed that all three circRNAs possessed potential 18S rRNA complementary sequences and SuRE elements (Fig. 1G). However, only hsa_circ_0008661 contained both the 40–60 nt SuRE element and the 18S rRNA complementary sequence, and the 18S rRNA complementary sequence was preferentially localized to the 5’ end of the SuRE element. Both circSLC25A24 and circYWHAZ do not have the appropriate structural features described above.

Then, a luciferase reporter plasmid was designed to quantitatively test IRES activity. Briefly, the putative IRESs of three circRNAs were inserted between Renillaluciferase (Rluc) and Luciferase (Luc) reporter genes, each of which has its own start and stop codons. The CMV promoter drove the expression of RLuc, and Luc was not expressed due to the absence of any promoter (Fig. 1H, upper). Only the IRES has putative activity, and Luc expression can be induced, which indicates that the IRES sequences can induce 5′-cap-independent translation [35, 36]. The dual-luciferase assay showed that the hsa_circ_0008661 IRES exhibited greater Luc/Rluc luciferase activity than the circSLC25A24 and circYWHAZ IRESs, suggesting that the IRES of hsa_circ_0008661 has a greater ability to induce 5′-cap-independent translation (Fig. 1H). Therefore, we focused on hsa_circ_0008661 in a subsequent study.

### Characterization of circCOPA in GBM

**Fig. 2:**
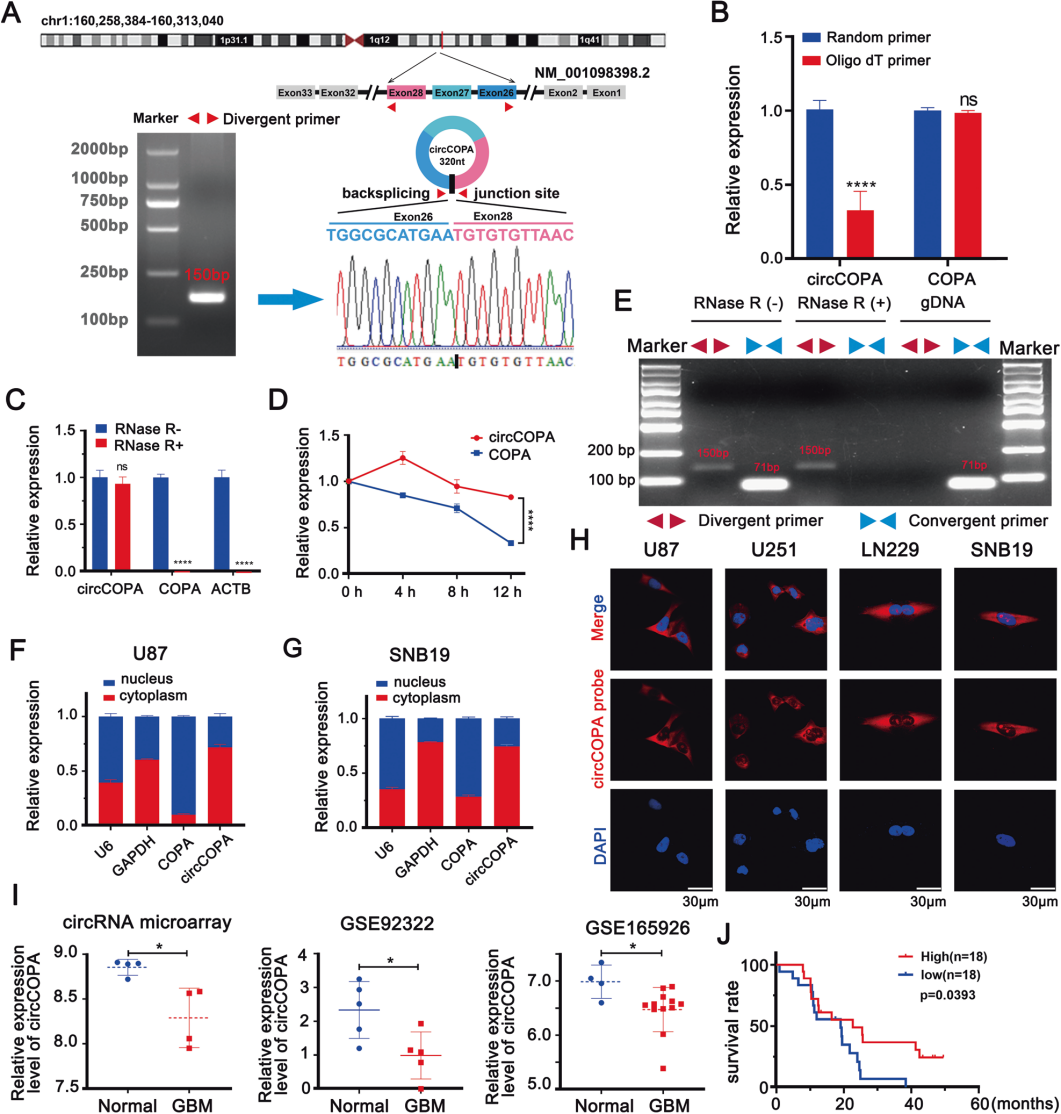
Characterization of circCOPA. **A** Illustration of circCOPA formation. CircCOPA originated from chromosome 1 (chr1:106,258,384-160,313,040) and was generated by back-splicing 26-28 exons from the *COPA* gene (NM_001098398.2). Sanger sequencing following PCR conducted using the divergent primers confirmed the junction site of circCOPA. **B** Relative expression levels of COPA mRNA and circCOPA in cDNA reverse transcribed with oligo dT primers and random primers (*n* = 3). **C** Relative RNA levels of COPA mRNA and circCOPA after RNase R treatment (*n* = 3). **D** Relative RNA levels of COPA mRNA and circCOPA at different time points after actinomycin D treatment (*n* = 3). **E** We designed divergent primers (red) and convergent primers (blue) to amplify the back-splicing or linear products from total RNA and genomic DNA (gDNA). Total RNA with or without RNase R treatment was subjected to PCR. **F**, **G** The expression levels of COPA mRNA and circCOPA were detected in the cytoplasmic and nuclear fractions of U87 and SNB19 cells. GAPDH and U6 RNA served as cytoplasmic and nuclear RNA markers, respectively. **H** FISH was used to detect the localization of circCOPA. Scale bar = 30 μm. **I** CircCOPA expression in our circRNA microarray dataset (Normal = 4 vs GBM = 4), the GSE92322 (Normal = 5 vs GBM = 5) and GSE165926 (Normal = 4 vs GBM = 11) datasets. **J** The relationship between circCOPA expression and patient prognosis in our clinical cohort (log-rank test). All data were shown as mean ± SD (bar plots). *p < 0.05, ****p < 0.0001, unpaired two-tailed Student’s *t*-test.

Based on the annotation in circBase, we found that hsa_circ_0008661 originates from chromosome 1 (chr1:106,258,384-160,313,040), is generated by back-splicing the 26–28 exons of the COPA gene (NM_001098398.2), and contains 320 nucleotides (Fig. 2A). According to the guidelines for naming eukaryotic circular RNAs [37], hsa_circ_0008661 was named as circCOPA (26,27,28), abbreviated as circCOPA.

To verify the circular structure of circCOPA, divergent primers specific for the back-splicing junction site of circCOPA for PCR were designed. Sanger sequencing of above PCR products confirmed the junction site of circCOPA (Fig. 2A). CircCOPA was amplified from random reverse-transcribed cDNA but not from oligo dT reverse-transcribed cDNA by using junction-specific primers, which confirmed its circular structure (Fig. 2B). Additionally, compared with COPA mRNA, circCOPA was more resistant to RNase R digestion and had a longer half-life (Fig. 2C, D). Next, a convergent primer that specifically amplified the canonical form of COPA was designed to further confirm the circular form of circCOPA (Fig. 2E). After reverse transcription with divergent primers (red), PCR was used to detect circCOPA, which is resistant to RNase R digestion. The divergent primers failed to amplify any products from genomic DNA, indicating that this outcome was not a result of genomic rearrangements or PCR artifacts. On the contrary, the COPA mRNA was amplified via convergent primers (blue). The PCR products disappeared after the total RNA was digested via RNase-R. Meanwhile, the cell fraction qPCR and FISH experiments revealed that circCOPA and COPA mRNA presented different cellular localizations in the U87, U251, SNB19 and LN229 cell lines. The majority of COPA mRNA was found in the nucleus, whereas circCOPA predominantly displayed cytoplasmic localization (Fig. 2F–H).

Finally, circCOPA expression in GBM tissues was analyzed. In our microarray dataset, circCOPA expression was lower in GBM tissues than that in paired normal brain tissues, which is consistent with the findings in the GEO92322 and GEO165926 datasets (Fig. 2I). Moreover, higher circCOPA expression was correlated with better patient prognosis in our clinical cohort (the median expression level was defined as the cutoff) (Fig. 2J). Collectively, these results reveal that circCOPA has a stable circular structure, is localized mainly to the cytoplasm, and is downregulated in GBM tissues.

### Tumor-suppressive effects of circCOPA

**Fig. 3:**
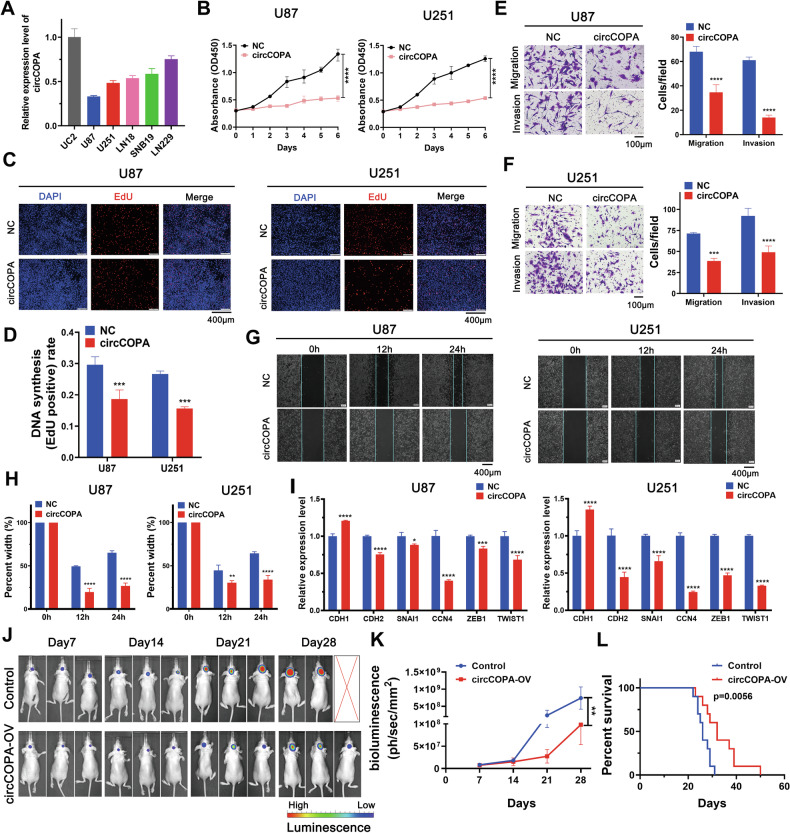
Tumor suppressive functions of circCOPA in GBM cell lines. **A** The expression level of circCOPA in GBM cells and UC2 astrocytes (*n* = 3). **B** CCK8 assays revealed that growth was inhibited in U87 and U251 cells overexpressing circCOPA (*n* = 3). **C**, **D** The effect of circCOPA overexpression on glioma proliferation was verified via EdU experiment in U87 and U251 cells (*n* = 3). Scale bar = 400 μm. **E**, **F** Transwell assay verified that the migration and invasion of U87 and U251 cells were limited after circCOPA overexpression (*n* = 3). Scale bar = 100 μm. **G**, **H** Wound healing assay found that the migration of U87 and U251 cells was reduced after circCOPA overexpression (*n* = 3). Scale bar = 400 μm. **I** CircCOPA overexpression promoted the expression of EMT-related CDH1 and inhibited the expression of EMT-related CDH2, CCN4, SNAI1, ZEB1 and TWIST1 in U87 and U251 cells (*n* = 3). EMT: epithelial-mesenchymal transition. Two-way ANOVA. **J** U87 cells with circCOPA overexpression and control cells were intracranially injected into nude mice. Representative bioluminescent images (at 7, 14, 21, and 28 days) of the glioma-bearing mice were obtained. **K** Bioluminescent intensity of glioma-bearing mice (at 7, 14, 21, and 28 days) (*n* = 5 for BALB/c nude mice). **L** Kaplan‒Meier survival curves of mice implanted with U87 cells (circCOPA-overexpressing cells vs. control cells, *p* = 0.0056, log-rank test). The data were shown as mean ± SD (bar plots). **p < 0.01, ***p < 0.001, ****p < 0.0001, unpaired two-tailed Student’s t test.

The circCOPA expression level in several GBM cell lines and UC2 astrocyte cells was measured via RT‒qPCR (Fig. 3A). CircCOPA was downregulated in GBM cell lines (U87, U251, LN118, SNB19 and LN229) compared with UC2 astrocyte cells. Additionally, the expression of circCOPA was lower in the U87 and U251 cell lines but greater in the SNB19 and LN229 cell lines. To further investigate the possible biological functions of circCOPA, a plasmid overexpressing circCOPA was constructed (Supplementary Fig. S1A). Moreover, three siRNAs targeting the circCOPA junction site were used to specifically knockdown circCOPA expression (Supplementary Fig. S1B). U87 and U251 cells were transfected with plasmids overexpressing circCOPA. Three siRNAs were separately transfected into LN229 and SNB19 cells. The efficiency and specificity of circCOPA knockdown and overexpression were verified by RT‒qPCR (Supplementary Fig. S1A, B). CircCOPA expression was increased in U87 and U251 cells, which were transfected with the circCOPA overexpression plasmid, but the expression of the host gene COPA did not change (Supplementary Fig. S1A). Notably, without affecting the expression of the host gene COPA, siRNA1 and siRNA2 decreased the expression of circCOPA in LN229 and SNB19 cells, while siRNA3 did not affect circCOPA expression (Supplementary Fig. S1B). Therefore, siRNA1 and siRNA2 were selected for subsequent experiments.

CircCOPA expression was lower in GBM samples than in paired normal brain samples (Fig. 2I), and we speculated that circCOPA may function as a tumor suppressor. CCK8 and EdU assays verified that the proliferation of U87 and U251 cells was notably limited after circCOPA upregulation (Fig. 3B–D), while the proliferation of LN229 and SNB19 cells was increased after circCOPA knockdown (Supplementary Fig. S1C–E). Transwell assays verified that the migration and invasion of U87 and U251 cells were limited after circCOPA overexpression (Fig. 3E, F). In contrast, knocking down circCOPA facilitated the migration and invasion of LN229 and SNB19 cells (Supplementary Fig. S1F, G). Moreover, in the wound healing assay, circCOPA overexpression reduced the migration of U87 and U251 cells (Fig. 3G, H), while circCOPA knockdown had the opposite effect on the migration of both LN229 and SNB19 cells (Supplementary Fig. S1H, I). RT-qPCR results further showed that circCOPA overexpression regulated the expression of EMT-related genes. Specifically, circCOPA overexpression promoted the expression of CDH1, but inhibited the expression of CDH2, CCN4, SNAI1, ZEB1 and TWIST1 in U87 and U251 cells (Fig. 3I). In vivo, circCOPA expression suppressed U87 cells intracranial tumor growth and increased the survival of the animals (Fig. 3J–L).

Taken together, our results suggest that circCOPA is downregulated in GBM tissues and cell lines, and circCOPA may have tumor-suppressive effects on GBM.

### CircCOPA encodes a novel protein, COPA-99aa

**Fig. 4:**
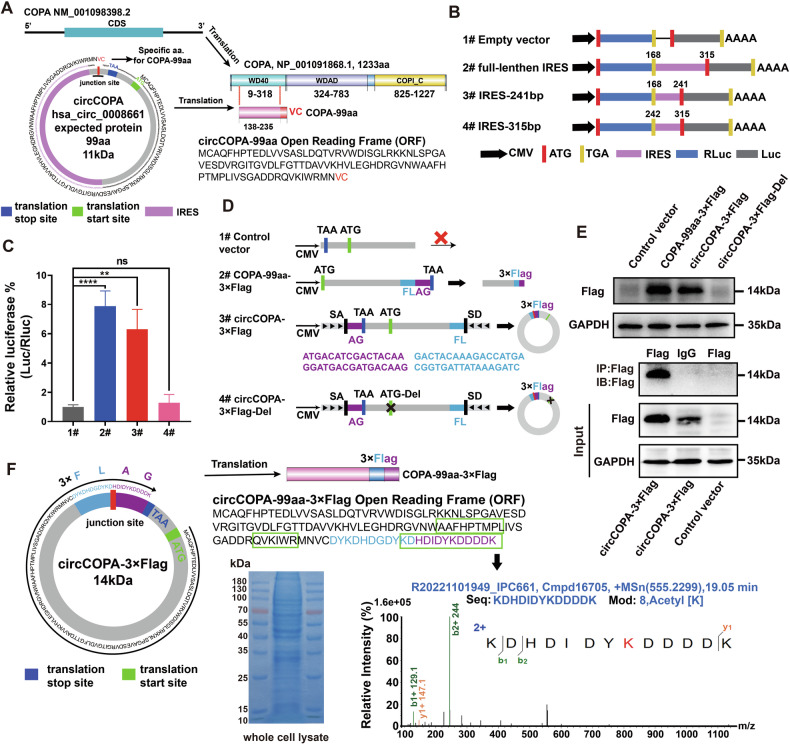
Identification of the ability of circCOPA to be encoded. **A** Illustration of the circCOPA-encoded protein and parental COPA protein. **B** The full-length IRES sequence in circCOPA or its different truncations were cloned between Rluc/Luc reporter genes. **C** The relative luciferase activity of Luc/Rluc was examined through a luciferase assay (*n* = 3). Data were shown as mean ± SD (bar plots). **p < 0.01, ****p < 0.0001, unpaired two-tailed Student’s *t*-test. **D** Illustration of plasmids used for detecting new proteins encoded by circCOPA. Control: the flanking sequences were deleted, which cannot form circCOPA. COPA-99aa-3×Flag: The linearized ORF of circCOPA with a 3×Flag tag was cloned and inserted into a plasmid and regarded as the positive control. CircCOPA-3×Flag: the junction site before the stop codon of the ORF was moved to the end of the sequence, and the 3×Flag sequence was separated on both sides, with flanking sequences, a splicing acceptor (SA) and a splicing donor (SD). The 3×Flag sequence was designed to cross the back-splicing junction site in circCOPA-3×Flag. CircCOPA-3×Flag-Del: lacking the start codon of the ORF. All vectors had a CMV promoter. **E** WB and IP assays using a Flag tag antibody were used to detect COPA-99aa expression in 293T cells transfected with the above plasmids. **F** Left panel: illustration of COPA-99aa-3×Flag encoded by circCOPA-3×Flag. Right panel: whole-cell lysates of 293T cells transfected with the circCOPA-3×Flag plasmid. The differential gel bands between 10–25 kDa were extracted for LC–MS analysis, and the COPA-99aa-3×Flag junction-specific sequence (KDHDIDYKDDDK) was identified.

CircCOPA has the potential to encode proteins and can inhibit the aggressive biological characteristics of GBM cells. Therefore, we further validated if circCOPA can encode a new protein or peptide. As shown in Fig. 4A, the host gene *COPA* (NM_001098398.2) encodes a 1233-amino-acid (aa) protein (NP_001091868.1). According to the annotation of circCOPA in TransCirc, circCOPA was able to translate a novel protein with 99 amino acids by using “ATG” as the start codon and “TAA” as the stop codon. The novel protein had a molecular weight of approximately 11 kDa and was named as COPA-99aa. COPA-99aa shares 97 amino acids in the WD40 domain of the parent protein (COPA). Additionally, the novel protein COPA-99aa has a unique C-terminus of “Val Cys (VC)” formed by the spanning junction open reading frame (ORF).

Our results revealed that the potential IRES (315 bp upstream of the ORF) of circCOPA had higher activity (Fig. 1H). To further verify which segment of the IRES has the main activity, we constructed a tandem Rluc–Luc reporter plasmid. The IRES-241bp, IRES-315bp and full-length IRES sequences were cloned between RLuc and Luc. Then, the relative luciferase activity (Luc/Rluc) was measured (Fig. 4B). A luciferase assay revealed that compared with the truncated IRES, the full-length IRES of circCOPA exhibited the highest Luc/Rluc activity (Fig. 4C). The luciferase activity of IRES-315bp was the lowest, similar to the empty vector, which suggested that the IRES-315bp did not stimulate ribosome entry. The luciferase activity of IRES-241bp was higher, which was close to that of the full-length IRES. These results indicate that IRES-241bp may mainly induce ribosome entry.

Then, a set of plasmids was constructed to verify whether circCOPA was translatable (Fig. 4D). A plasmid with deleted flanking sequences was used as a control. The linearized ORF with a 3×Flag tag was inserted into a plasmid (COPA-99aa-3×Flag) and used as a positive control. We moved the junction site before the stop codon of the ORF to the end of the sequence and separated the 3×Flag sequence on both sides, with flanking sequences as well as the SA and SD sequences (circCOPA-3×Flag). Notably, as the 3×Flag sequence was designed to cross the back-splicing junction site in circCOPA-3×Flag, COPA-99aa-3×Flag has a special Flag tag formed by the “spanning Flag junction ORF”. Additionally, a plasmid lacking the start codon of the ORF was constructed (circCOPA-3×Flag-Del). These plasmids were separately transfected into 293T cells and their potential translated products were detected. Immunoblotting revealed that the Flag tag antibody could detect an approximately 14 kDa protein in COPA-99aa-3×Flag- and circCOPA-3×Flag-transfected cells but not in circCOPA-3×Flag-Del-transfected cells (Fig. 4E, upper). Moreover, an IP assay further confirmed the presence of the predicted protein in COPA-99aa-3×Flag-transfected cells by using a Flag tag antibody (Fig. 4E, lower).

Importantly, via LC‒MS/MS analysis, we detected the amino acid sequence (KDHDIDKDDDDK) of a special Flag tag formed by the “spanning Flag junction ORF” in circCOPA-3×Flag-transfected 293T cells (Fig. 4F, Supplementary Fig. S2A). Another two sequences of COPA-99aa were also identified (Supplementary Fig. S2B, C). Collectively, these results verify that circCOPA can encode the novel protein COPA-99aa through its IRES sequence.

### COPA-99aa, but not circCOPA, inhibits the GBM malignant phenotype

**Fig. 5:**
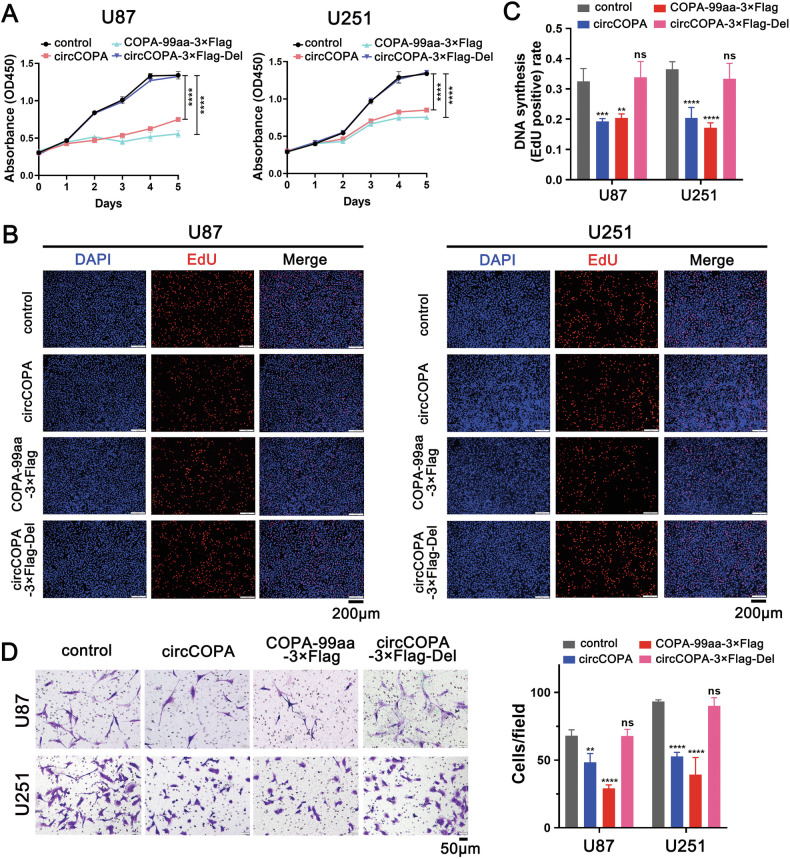
CircCOPA inhibited the GBM malignant phenotype by encoding COPA-99aa. CCK8 (**A**), EdU (**B**, **C**) and Transwell (**D**) assays revealed that circCOPA limited the growth and migration of U87 and U251 cells by encoding COPA-99aa (*n* = 3). All data were shown as mean ± SD (bar plots). **B**, Scale bar = 200 μm. **D** Scale bar = 50 μm. **p < 0.01, ****p < 0.0001, unpaired two-tailed Student’s *t*-test.

A previous study reported that the protein/peptide, but not the circRNA itself, inhibited the aggressive biological characteristics of GBM [38]. Therefore, we also investigated whether circCOPA exerted its biological function through its encoded protein (COPA-99aa). U87 and U251 cells were separately transfected with the control, circCOPA (Supplementary Fig. S1A, upper), COPA-99aa-3×Flag (Fig. 4D, 3#), circCOPA-3×Flag-Del (Fig. 4D, 4#) plasmids. Then, the biological functions of COPA-99aa in U87 and U251 cells were further analyzed. CCK8 and EdU assays verified that the proliferation of U87 and U251 cells, which were transfected with the COPA-99aa-3×Flag and circCOPA plasmids, was inhibited (Fig. 5A–C). In contrast, the circCOPA-3×Flag-Del overexpression plasmid failed to affect the proliferation of U87 or U251 cells (Fig. 5A–C). Moreover, a transwell assay indicated that migration was limited in U87 and U251 cells transfected with COPA-99aa-3×Flag and circCOPA plasmid but not in U87 and U251 cells transfected with circCOPA-3×Flag-Del plasmid (Fig. 5D). These results prove that COPA-99aa, but not circCOPA, inhibits the GBM malignant phenotype.

### COPA-99aa disrupts the binding of NONO and SFPQ

To clarify the mechanism of COPA-99aa in GBM, we first conducted RNA-seq analysis of U87 cells transfected with COPA-99aa-3×Flag and control plasmid. We screened 336 differentially expressed genes (DEGs) (Supplementary Fig. S3A, B). KEGG enrichment results revealed that DEGs were associated with ferroptosis, the Hippo signaling pathway, the JAK-STAT signaling pathway, the Wnt signaling pathway, and breast cancer (Supplementary Fig. S3C). GO analysis suggested that DEGs were mainly related with cell differentiation, cellular response to chemical stimulus, and negative regulation of biological process (Supplementary Fig. S3D). Disease ontology (DO) analysis revealed that these DEGs were related to many types of cancers (Supplementary Fig. S3E). These results suggest that COPA-99aa may function as a regulator of GBM occurrence, progression, and chemosensitivity.

**Fig. 6:**
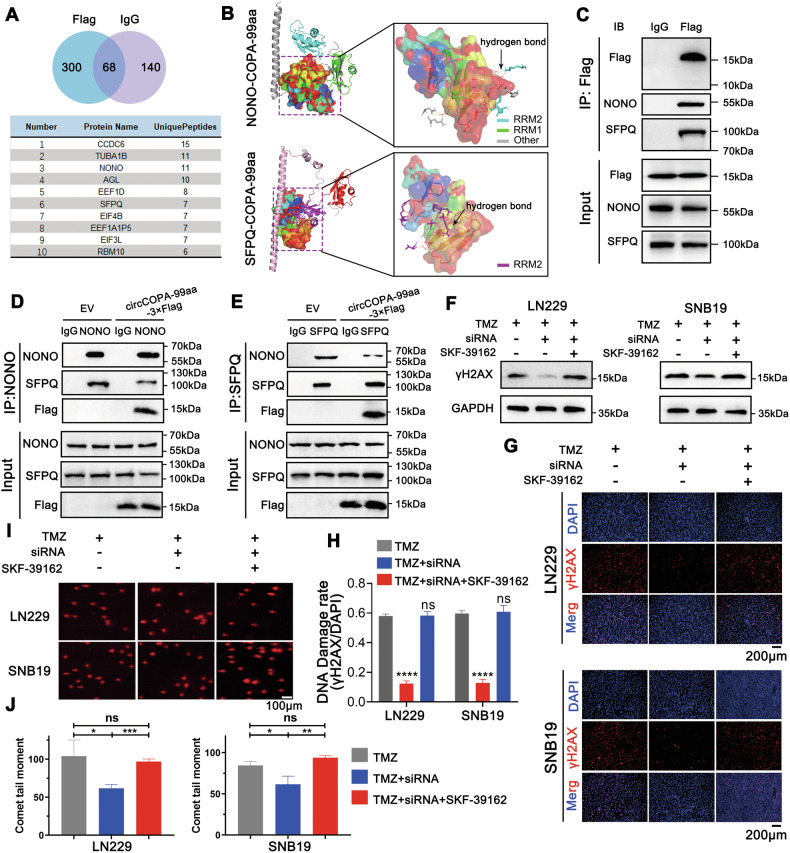
COPA-99aa disrupts the NONO–SFPQ complex to increase DNA damage in GBM cells. **A** IP and LC–MS/MS analyses identified 300 protein candidates that may specifically interact with COPA-99aa. The top 10 protein candidates ordered by the quantity of unique peptides are displayed. **B** Molecular docking simulations revealed that the NONO and SFPQ proteins bound to COPA-99aa through hydrogen bond interactions. The result was modified by using PyMol. **C** IP confirmed that COPA-99aa could bind to the NONO and SFPQ proteins. **D**, **E** Co-IP revealed that the mutual interaction between NONO and SFPQ was weakened in 293T cells transfected with circCOPA-Flag. WB (**F**) and IF (**G**, **H**) assays revealed that circCOPA silencing reduced the expression of a DNA damage marker (γH2AX) in GBM cells treated with TMZ (*n* = 3). Simultaneous disruption of the NONO–SFPQ complex by using auranofin (SKF-39162) reversed the decrease in γH2AX. Scale bar = 100 μm. **I**, **J** Comet assay showed that the suppression of circCOPA reduced the extent of DNA damage in GBM cells treated with TMZ (*n* = 3). Simultaneous disruption of the NONO–SFPQ complex by using SKF-39162 reversed the decrease in the extent of DNA damage. The comet tail moment was used to measure the level of DNA damage. Scale bar = 100 μm. All data were shown as mean ± SD (bar plots). *p < 0.05, **p < 0.01, ***p < 0.001, ****p < 0.0001, unpaired two-tailed Student’s *t*-test.

We further performed LC–MS/MS analysis to identify COPA-99aa-interacting protein candidates. Three hundred protein candidates that specifically interact with COPA-99aa were identified (Fig. 6A). Among the top 10 protein candidates ordered by the quantity of unique peptides, 11 unique peptides of NONO and 7 unique peptides of SFPQ were identified (Fig. 6A, Supplementary Fig. S4A, B). Importantly, NONO and SFPQ can form a complex to promote DSB repair via the NHEJ pathway in the nucleus [22, 39, 40]. Additionally, the expression of NONO in GBM is increased and can promote the occurrence, growth and invasion of glioma [41, 42]. Our results also showed that COPA-99aa was not only distributed in the cytoplasm, but also localized in the nucleus in U87, U251, LN229 and SNB19 cells (Supplementary Fig. S4C). Thus, we focused on exploring the relationship between COPA-99aa and the NONO–SFPQ complex.

The predicted amino acid sequence of COPA-99aa shared 97 amino acids in the WD40 domain of the parental protein COPA (Fig. 4A). We predicted the three-dimensional (3D) structure of COPA-99aa through the I-TASSER service (Supplementary Fig. S5A). Both NONO and SFPQ encompasses the DBHS domain (including RRM1, RRM2, NOPS, and an extensive coiled-coil region) [24, 25] (Supplementary Fig. S5B, C). Molecular docking simulations of COPA-99aa to NONO and SFPQ were performed via the Cluspro 2.0 server. Docking simulation revealed that the RRM1 and RRM2 domains of NONO could separately bind to COPA-99aa through hydrogen bond interactions (Fig. 6B). Moreover, the RRM2 domain of SFPQ could interact with COPA-99aa via hydrogen bond interactions (Fig. 6B). Co-IP assays verified the interaction between COPA-99aa and the NONO–SFPQ complex. When the Flag antibody was used to immunoprecipitate target proteins, COPA-99aa-Flag successfully pulled down NONO in cells transfected with the circCOPA-Flag plasmid (Fig. 6C). Additionally, SFPQ was pulled down by COPA-99aa-Flag (Fig. 6C). Therefore, COPA-99aa can interact with the NONO–SFPQ complex.

One study reported that the RRM1 domain is typically essential for RNA binding. The coiled-coil, RRM2, and NOPS are responsible for the dimerization and stability of the NONO–SFPQ complex [25, 26]. Given the results from the Co-IP assay and docking simulation (Fig. 6B, C), which indicated that COPA-99aa interacted with NONO and SFPQ, respectively, we further characterized the influence of COPA-99aa on the interaction between NONO and SFPQ. NONO bound to smaller quantities of SFPQ when COPA-99aa was overexpressed (Fig. 6D). Moreover, less NONO was pulled down by SFPQ when COPA-99aa was overexpressed (Fig. 6E). As mentioned earlier, the NONO and SFPQ proteins are mainly dimerized by RRM2 and NOPS domains. Therefore, these results suggest that COPA-99aa may interfere with the dimerization of NONO and SFPQ by binding to their RRM2 domains, thereby disrupting their function.

### COPA-99aa inhibits the proliferation and invasion and increases DNA damage in GBM cells by disrupting the dimerization of NONO and SFPQ

To explore the effect of the interaction between COPA-99aa and NONO–SFPQ complex on GBM cells, the potential drug auranofin (SKF-39162), which can disrupt the formation of the NONO–SFPQ complex by degrading the NONO protein, was screened [41]. CCK8 assay showed that knockdown of circCOPA promoted the proliferation of LN229 and SNB19 cells, but simultaneous disruption of the NONO–SFPQ complex partly reversed their growth (Supplementary Fig. S5D, E). Transwell assay indicated that circCOPA silencing stimulated the invasion of LN229 and SNB19 cells, but the increase of invasion was partly eliminated after simultaneous disruption of the NONO–SFPQ complex (Supplementary Fig. S5F).

The NONO–SFPQ complex can promote DSB repair [22, 39], and TMZ is the primary chemotherapeutic agent used for GBM treatment by inducing DNA DSB [43]. Thus, we attempted to explore whether COPA-99aa affects NONO–SFPQ complex-mediated DNA damage repair during TMZ treatment. First, WB and IF assays verified that circCOPA overexpression promoted the expression of a DNA damage marker (γH2AX) caused by TMZ treatment (Supplementary Fig. S5G, H). Comet assays revealed that DNA damage induced by TMZ was increased after overexpression of circCOPA (Supplementary Fig. S5I). Moreover, WB and IF assays also revealed that when circCOPA was knocked down, γH2AX caused by TMZ treatment was reduced, and simultaneous disruption of the NONO–SFPQ complex reversed the decrease in γH2AX (Fig. 6F–H). Comet assays further revealed that DNA damage induced by TMZ was weakened after the inhibition of circCOPA, and the level of DNA damage was reversed after simultaneous disruption of the NONO–SFPQ complex (Fig. 6I, J). In summary, these results suggest that COPA-99aa can inhibit the proliferation and invasion, and increase TMZ-induced DNA damage by interfering with the dimerization of NONO and SFPQ in GBM cells.

## Discussion

GBM is a highly aggressive and malignant primary brain tumor. Despite advanced treatments, the prognosis of GBM patients remains unsatisfactory, with a median survival time of <2 years [44]. Exploring the molecular and pathological mechanism of GBM is expected to provide a new strategy for the treatment of GBM patients [45]. Numerous studies have confirmed the importance of circRNAs in the molecular and pathological mechanisms of GBM [7, 13]. CircRNAs can regulate the proliferation, invasion, angiogenesis, tumor microenvironment and therapeutic resistance of GBM by sponging miRNAs or binding to RNA binding proteins [13]. Importantly, circRNAs can encode novel peptides/proteins through IRES, N6-methyladenosine (m6A) modification and an infinite number of open reading frames. These novel peptides/proteins play key roles in the tumorigenesis, growth and treatment resistance of GBM and are associated with patient prognosis. Zhong et al. reported that m6A-modified circMET is highly expressed in GBM and can encode a MET variant (MET404), whose high expression predicts poor prognosis in GBM patients [12]. Gao et al. reported that circ-E-Cad encodes a novel protein C-E-Cad in GBM, which promotes the tumorigenicity of glioma stem cell [33]. Inhibition of C-E-Cad enhances the sensitivity of GBM to anti-EGFR therapy. CircRNAs encoding peptides/proteins are considered as promising targets and molecular biomarkers for glioma therapy.

Using a circRNA microarray, our study revealed that the expression of circCOPA was lower in GBM tissues than that in adjacent normal brain tissues (Fig. 1A). CircCOPA functioned as a suppressor of the GBM malignant phenotype, including the proliferation, migration and invasion of GBM cells (Figs. 2, and 3). In vivo, circCOPA expression suppressed U87 cells intracranial tumor growth and increased the survival of the animals (Fig. 3I). Higher circCOPA expression was correlated with better patient prognosis in our clinical cohort (Fig. 2J). Given the important function of circRNA-encoded proteins, we further validated the encoding ability of circCOPA. CircCOPA was able to encode the novel protein COPA-99aa through its IRES (Fig. 4). Interestingly, our study revealed that circCOPA mainly inhibited the malignant behaviors of GBM cells by encoding COPA-99aa (Fig. 5). Thus far, these results indicate that circCOPA and its encoded COPA-99aa play a crucial role in suppressing the occurrence and development of GBM, and further investigation is needed to uncover their potential molecular mechanisms.

The NONO–SFPQ complex is a heterodimer and is a crucial effector of DNA damage repair, alternative splicing and RNA binding [46]. SFPQ and NONO serve as regulators at telomeres, collaborating to maintain telomere integrity by inhibiting telomere fragility and homologous recombination [47]. In triple-negative breast cancer, NONO and SFPQ regulate DSB repair in a PARP-dependent manner [22]. Both NONO and SFPQ commonly contain RRM1, RRM2, and NOPS domains and a part of the coiled-coil. RRM2 and NOPS mainly lead to the dimerization of NONO and SFPQ, which are required to stabilize protein structure and function. Fu et al. reported that the lncRNA NPPA-AS1 competitively interacts with SFPQ due to the overlapping binding sites of SFPQ and NONO, thus increasing DNA damage in cardiomyocyte cells from mice with myocardial infarction [20]. LC–MS/MS analysis, molecular docking simulations and co-IP assays also revealed that COPA-99aa can bind to NONO and SFPQ (Fig. 6A–C). Interestingly, docking simulations showed that COPA-99aa can bind to the RRM2 domains of NONO and SFPQ, respectively (Fig. 6B). Co-IP further confirmed that COPA-99aa can interact with both NONO and SFPQ, thus interfering with their dimerization (Fig. 6D, E).

**Fig. 7:**
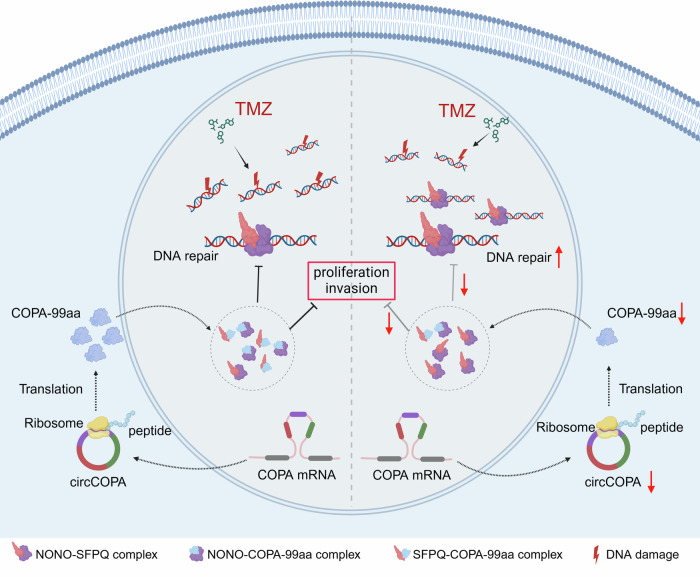
Summary figure of the mechanism of circCOPA. CircCOPA encodes the COPA-99aa protein through its IRES. COPA-99aa can interact with NONO and SFPQ proteins to disrupt their dimerization, which inhibits proliferation, invasion and the DNA damage repair in GBM cells. However, when circCOPA is downregulated, the COPA-99aa protein is consequently decreased. Then, the dimerization of NONO and SFPQ is no longer limited, resulting in the increase of DNA damage repair. GBM cells become insensitive to TMZ. Meanwhile, the inhibitory ability of COPA-99aa on GBM cell proliferation and invasion is weakened. Created with BioRender.com.

Moreover, the NONO–SFPQ complex participates in the NHEJ pathway and can promote DSB repair [39]. For example, Krietsch et al. revealed that the attenuation of NONO decelerates the end joining reaction in irradiation-treated HeLa cells, promoting cell death [48]. Thus, we further investigated whether COPA-99aa can inhibit the DSB repair ability of the NONO–SFPQ complex after TMZ treatment in GBM cells. Our results revealed that inhibition of circCOPA reduced γH2AX expression caused by TMZ treatment (Fig. 6F–H). Simultaneous disruption of the NONO–SFPQ complex reversed the decrease in γH2AX. Comet assays also revealed that DNA damage was weakened after circCOPA inhibition, which was reversed after simultaneous disruption of the NONO–SFPQ complex (Fig. 6I, J). Additionally, some groups reported that NONO and SFPQ can promote the proliferation and invasion of cancer cells [49, 50]. Our results verified that circCOPA silencing promoted the proliferation and invasion of LN229 and SNB19 cells, but simultaneous disruption of the NONO–SFPQ complex partly reversed their growth and invasion. Collectively, these results suggest that COPA-99aa disrupts the dimerization of NONO and SFPQ, thus suppressing the proliferation and invasion, and increasing the DNA damage caused by TMZ treatment in GBM cells (Fig. 7).

However, our study also has certain limitations. CircRNA biogenesis is regulated by multiple effectors, including m6A modifications, trans-factors, and cis-elements [7]. Further investigation is required to delve into the mechanisms responsible for the reduced expression of circCOPA in GBM. Additionally, a larger cohort is needed to further test the circCOPA expression level to confirm whether circCOPA can serve as an independent biomarker for GBM. The development of specific antibodies against COPA-99aa is also needed for the analysis of its clinical features. Mechanistically, in addition to its role in DNA damage repair, the NONO–SFPQ complex is also involved in alternative splicing and RNA binding [51]. Whether COPA-99aa affects alternative splicing and RNA binding in GBM by interfering with the dimerization of the NONO–SFPQ complex needs to be further investigated.

## Conclusion

The expression of circCOPA is downregulated in GBM tissues and is positively associated with better prognosis in GBM patients. CircCOPA is able to encode a unique protein COPA-99aa, which can suppress the ability of proliferation, migration and invasion of GBM cells. COPA-99aa can increase the DNA damage caused by TMZ treatment by disrupting the dimerization of the NONO–SFPQ complex. Overall, circCOPA mainly inhibits the GBM malignant phenotype via the encoded COPA-99aa. COPA-99aa suppresses the proliferation and invasion, and increases TMZ-induced DNA damage by disrupting the NONO–SFPQ complex. These results suggest that restoring the balance of circCOPA or COPA-99aa expression can help improve or maintain the efficacy of TMZ treatment in GBM patients.

### Supplementary information

Supplementary materials

Supplementary Table 1

Supplementary Table 2

Original western blots

## Data Availability

The datasets used and/or analyzed during the current study are available from the corresponding author on reasonable request.

## References

[1] Lowe SR, Kunigelis K, Vogelbaum MA. Leveraging the neurosurgical operating room for therapeutic development in NeuroOncology. Adv drug Deliv Rev. 2022;186:114337. 35561836 10.1016/j.addr.2022.114337

[2] Louis DN, Perry A, Wesseling P, Brat DJ, Cree IA, Figarella-Branger D, et al. The 2021 WHO classification of tumors of the central nervous system: a summary. Neuro Oncol. 2021;23:1231–51. 34185076 10.1093/neuonc/noab106 PMC8328013

[3] McFaline-Figueroa JR, Lee EQ. Brain Tumors. Am J Med. 2018;131:874–82. 29371158 10.1016/j.amjmed.2017.12.039

[4] Stupp R, Taillibert S, Kanner A, Read W, Steinberg D, Lhermitte B, et al. Effect of tumor-treating fields plus maintenance temozolomide vs maintenance temozolomide alone on survival in patients with glioblastoma: a randomized clinical trial. JAMA. 2017;318:2306–16. 29260225 10.1001/jama.2017.18718 PMC5820703

[5] Long S, Huang G, Ouyang M, Xiao K, Zhou H, Hou A, et al. Epigenetically modified AP-2α by DNA methyltransferase facilitates glioma immune evasion by upregulating PD-L1 expression. Cell Death Dis. 2023;14:365. 37330579 10.1038/s41419-023-05878-x PMC10276877

[6] Guan L, Hao Q, Shi F, Gao B, Wang M, Zhou X, et al. Regulation of the tumor immune microenvironment by cancer-derived circular RNAs. Cell Death Dis. 2023;14:132. 36797245 10.1038/s41419-023-05647-w PMC9935907

[7] Peng D, Luo L, Zhang X, Wei C, Zhang Z, Han L. CircRNA: An emerging star in the progression of glioma. Biomed Pharmacother. 2022;151:113150. 35623170 10.1016/j.biopha.2022.113150

[8] Vo JN, Cieslik M, Zhang Y, Shukla S, Xiao L, Zhang Y, et al. The landscape of circular RNA in cancer. Cell. 2019;176:869–81.e813. 30735636 10.1016/j.cell.2018.12.021 PMC6601354

[9] Mu M, Niu W, Chu F, Dong Q, Hu S, Niu C. CircSOBP suppresses the progression of glioma by disrupting glycolysis and promoting the MDA5-mediated immune response. iScience. 2023;26:107897. 37766977 10.1016/j.isci.2023.107897 PMC10520879

[10] Geng X, Zhang Y, Lin X, Zeng Z, Hu J, Hao L, et al. Exosomal circWDR62 promotes temozolomide resistance and malignant progression through regulation of the miR-370-3p/MGMT axis in glioma. Cell Death Dis. 2022;13:596. 35817771 10.1038/s41419-022-05056-5 PMC9273787

[11] Gao X, Xia X, Li F, Zhang M, Zhou H, Wu X, et al. Circular RNA-encoded oncogenic E-cadherin variant promotes glioblastoma tumorigenicity through activation of EGFR-STAT3 signalling. Nat Cell Biol. 2021;23:278–91. 33664496 10.1038/s41556-021-00639-4

[12] Zhong J, Wu X, Gao Y, Chen J, Zhang M, Zhou H, et al. Circular RNA encoded MET variant promotes glioblastoma tumorigenesis. Nat Commun. 2023;14:4467. 37491377 10.1038/s41467-023-40212-1 PMC10368723

[13] Liu CX, Chen LL. Circular RNAs: Characterization, cellular roles, and applications. Cell. 2022;185:2016–34. 35584701 10.1016/j.cell.2022.04.021

[14] Qu L, Yi Z, Shen Y, Lin L, Chen F, Xu Y, et al. Circular RNA vaccines against SARS-CoV-2 and emerging variants. Cell. 2022;185:1728–44.e1716. 35460644 10.1016/j.cell.2022.03.044 PMC8971115

[15] Ruan X, Liu Y, Wang P, Liu L, Ma T, Xue Y, et al. RBMS3-induced circHECTD1 encoded a novel protein to suppress the vasculogenic mimicry formation in glioblastoma multiforme. Cell Death Dis. 2023;14:745. 37968257 10.1038/s41419-023-06269-y PMC10651854

[16] Chen Y, Mu Y, Guan Q, Li C, Zhang Y, Xu Y, et al. RPL22L1, a novel candidate oncogene promotes temozolomide resistance by activating STAT3 in glioblastoma. Cell Death Dis. 2023;14:757. 37985768 10.1038/s41419-023-06156-6 PMC10662465

[17] Maugeri-Saccà M, Bartucci M, De Maria R. DNA damage repair pathways in cancer stem cells. Mol Cancer Ther. 2012;11:1627–36. 22844074 10.1158/1535-7163.MCT-11-1040

[18] Zhang WW, Zhang LX, Busch RK, Farrés J, Busch H. Purification and characterization of a DNA-binding heterodimer of 52 and 100 kDa from HeLa cells. Biochem J. 1993;290:267–72. 8439294 10.1042/bj2900267 PMC1132410

[19] Schell B, Legrand P, Fribourg S. Crystal structure of SFPQ-NONO heterodimer. Biochimie. 2022;198:1–7. 35245601 10.1016/j.biochi.2022.02.011

[20] Fu W, Ren H, Shou J, Liao Q, Li L, Shi Y, et al. Loss of NPPA-AS1 promotes heart regeneration by stabilizing SFPQ-NONO heteromer-induced DNA repair. Basic Res Cardiol. 2022;117:10. 35247074 10.1007/s00395-022-00921-y

[21] Qiu M, Zhang N, Yao S, Zhou H, Chen X, Jia Y, et al. DNMT3A-mediated high expression of circ_0057504 promotes benzo[a]pyrene-induced DNA damage via the NONO-SFPQ complex in human bronchial epithelial cells. Environ Int. 2022;170:107627. 36399942 10.1016/j.envint.2022.107627

[22] de Silva HC, Lin MZ, Phillips L, Martin JL, Baxter RC. IGFBP-3 interacts with NONO and SFPQ in PARP-dependent DNA damage repair in triple-negative breast cancer. Cell Mol Life Sci. 2019;76:2015–30. 30725116 10.1007/s00018-019-03033-4 PMC11105386

[23] Li S, Li Z, Shu FJ, Xiong H, Phillips AC, Dynan WS. Double-strand break repair deficiency in NONO knockout murine embryonic fibroblasts and compensation by spontaneous upregulation of the PSPC1 paralog. Nucleic Acids Res. 2014;42:9771–80. 25100870 10.1093/nar/gku650 PMC4150768

[24] Lee M, Sadowska A, Bekere I, Ho D, Gully BS, Lu Y, et al. The structure of human SFPQ reveals a coiled-coil mediated polymer essential for functional aggregation in gene regulation. Nucleic Acids Res. 2015;43:3826–40. 25765647 10.1093/nar/gkv156 PMC4402515

[25] Laurenzi T, Palazzolo L, Taiana E, Saporiti S, Ben Mariem O, Guerrini U, et al. Molecular modelling of NONO and SFPQ dimerization process and RNA recognition mechanism. Int J Mol Sci. 2022;23:7626. 35886974 10.3390/ijms23147626 PMC9324803

[26] Passon DM, Lee M, Rackham O, Stanley WA, Sadowska A, Filipovska A, et al. Structure of the heterodimer of human NONO and paraspeckle protein component 1 and analysis of its role in subnuclear body formation. Proc Natl Acad Sci USA 2012;109:4846–50. 22416126 10.1073/pnas.1120792109 PMC3324020

[27] Zhou X, Wang R, Li X, Yu L, Hua D, Sun C, et al. Splicing factor SRSF1 promotes gliomagenesis via oncogenic splice-switching of MYO1B. J Clin Investig. 2019;129:676–93. 30481162 10.1172/JCI120279 PMC6355305

[28] Li Y, Chen J, Chen Z, Xu X, Weng J, Zhang Y, et al. CircGLIS3 promotes high-grade glioma invasion via modulating ezrin phosphorylation. Front Cell Dev Biol. 2021;9:663207. 34540823 10.3389/fcell.2021.663207 PMC8446459

[29] Liu M, Wang Q, Shen J, Yang BB, Ding X. Circbank: a comprehensive database for circRNA with standard nomenclature. RNA Biol. 2019;16:899–905. 31023147 10.1080/15476286.2019.1600395 PMC6546381

[30] Chen X, Han P, Zhou T, Guo X, Song X, Li Y. circRNADb: a comprehensive database for human circular RNAs with protein-coding annotations. Sci Rep. 2016;6:34985. 27725737 10.1038/srep34985 PMC5057092

[31] Huang W, Ling Y, Zhang S, Xia Q, Cao R, Fan X, et al. TransCirc: an interactive database for translatable circular RNAs based on multi-omics evidence. Nucleic Acids Res. 2021;49:D236–42. 33074314 10.1093/nar/gkaa823 PMC7778967

[32] Wei C, Zhang X, Peng D, Zhang X, Guo H, Lu Y, et al. LncRNA HOXA11-AS promotes glioma malignant phenotypes and reduces its sensitivity to ROS via Tpl2-MEK1/2-ERK1/2 pathway. Cell Death Dis. 2022;13:942. 36351895 10.1038/s41419-022-05393-5 PMC9646708

[33] Zhong J, Yang X, Chen J, He K, Gao X, Wu X, et al. Circular EZH2-encoded EZH2-92aa mediates immune evasion in glioblastoma via inhibition of surface NKG2D ligands. Nat Commun. 2022;13:4795. 35970825 10.1038/s41467-022-32311-2 PMC9378736

[34] Chen CK, Cheng R, Demeter J, Chen J, Weingarten-Gabbay S, Jiang L, et al. Structured elements drive extensive circular RNA translation. Mol Cell. 2021;81:4300–18.e4313. 34437836 10.1016/j.molcel.2021.07.042 PMC8567535

[35] Yang Y, Wang Z. IRES-mediated cap-independent translation, a path leading to hidden proteome. J Mol Cell Biol. 2019;11:911–9. 31504667 10.1093/jmcb/mjz091 PMC6884710

[36] Zhang M, Huang N, Yang X, Luo J, Yan S, Xiao F, et al. A novel protein encoded by the circular form of the SHPRH gene suppresses glioma tumorigenesis. Oncogene. 2018;37:1805–14. 29343848 10.1038/s41388-017-0019-9

[37] Chen LL, Bindereif A, Bozzoni I, Chang HY, Matera AG, Gorospe M, et al. A guide to naming eukaryotic circular RNAs. Nat cell Biol. 2023;25:1–5. 36658223 10.1038/s41556-022-01066-9 PMC10114414

[38] Xia X, Li X, Li F, Wu X, Zhang M, Zhou H, et al. A novel tumor suppressor protein encoded by circular AKT3 RNA inhibits glioblastoma tumorigenicity by competing with active phosphoinositide-dependent Kinase-1. Mol Cancer. 2019;18:131. 31470874 10.1186/s12943-019-1056-5 PMC6716823

[39] Jaafar L, Li Z, Li S, Dynan WS. SFPQ•NONO and XLF function separately and together to promote DNA double-strand break repair via canonical nonhomologous end joining. Nucleic Acids Res. 2017;45:1848–59. 27924002 10.1093/nar/gkw1209 PMC5605232

[40] Rajesh C, Baker DK, Pierce AJ, Pittman DL. The splicing-factor related protein SFPQ/PSF interacts with RAD51D and is necessary for homology-directed repair and sister chromatid cohesion. Nucleic Acids Res. 2011;39:132–45. 20813759 10.1093/nar/gkq738 PMC3017596

[41] Wang X, Han M, Wang S, Sun Y, Zhao W, Xue Z, et al. Targeting the splicing factor NONO inhibits GBM progression through GPX1 intron retention. Theranostics. 2022;12:5451–69. 35910786 10.7150/thno.72248 PMC9330516

[42] Wei Y, Luo H, Yee PP, Zhang L, Liu Z, Zheng H, et al. Paraspeckle protein NONO promotes TAZ phase separation in the nucleus to drive the oncogenic transcriptional program. Adv Sci. 2021;8:e2102653. 10.1002/advs.202102653 PMC8693076 34716691

[43] Stupp R, Mason WP, van den Bent MJ, Weller M, Fisher B, Taphoorn MJ, et al. Radiotherapy plus concomitant and adjuvant temozolomide for glioblastoma. N Engl J Med. 2005;352:987–96. 15758009 10.1056/NEJMoa043330

[44] Tan AC, Ashley DM, López GY, Malinzak M, Friedman HS, Khasraw M. Management of glioblastoma: state of the art and future directions. CA Cancer J Clinic. 2020;70:299–312. 10.3322/caac.21613 32478924

[45] Jiang T, Nam DH, Ram Z, Poon WS, Wang J, Boldbaatar D, et al. Clinical practice guidelines for the management of adult diffuse gliomas. Cancer Lett. 2021;499:60–72. 33166616 10.1016/j.canlet.2020.10.050

[46] Yu D, Huang CJ, Tucker HO. Established and evolving roles of the multifunctional Non-POU domain-containing octamer-binding protein (NonO) and splicing factor proline- and glutamine-rich (SFPQ). J Dev Biol. 2024;12:3. 38248868 10.3390/jdb12010003 PMC10801543

[47] Petti E, Buemi V, Zappone A, Schillaci O, Broccia PV, Dinami R, et al. SFPQ and NONO suppress RNA:DNA-hybrid-related telomere instability. Nat Commun. 2019;10:1001. 30824709 10.1038/s41467-019-08863-1 PMC6397292

[48] Krietsch J, Caron MC, Gagné JP, Ethier C, Vignard J, Vincent M, et al. PARP activation regulates the RNA-binding protein NONO in the DNA damage response to DNA double-strand breaks. Nucleic Acids Res. 2012;40:10287–301. 22941645 10.1093/nar/gks798 PMC3488241

[49] Lone BA, Siraj F, Sharma I, Verma S, Karna SKL, Ahmad F, et al. Non-POU domain-containing octomer-binding (NONO) protein expression and stability promotes the tumorigenicity and activation of Akt/MAPK/β-catenin pathways in human breast cancer cells. Cell Commun Signal. 2023;21:157. 37370134 10.1186/s12964-023-01179-0 PMC10294335

[50] Cheng Z, Lu C, Wang H, Wang N, Cui S, Yu C, et al. Long noncoding RNA LHFPL3-AS2 suppresses metastasis of non-small cell lung cancer by interacting with SFPQ to regulate TXNIP expression. Cancer Lett. 2022;531:1–13. 35101541 10.1016/j.canlet.2022.01.031

[51] Yarosh CA, Iacona JR, Lutz CS, Lynch KW. PSF: nuclear busy-body or nuclear facilitator? Wiley Interdiscip Rev RNA. 2015;6:351–67. 25832716 10.1002/wrna.1280 PMC4478221

