# Supplementary material for: A novel protein encoded by circCOPA inhibits the malignant phenotype of glioblastoma cells and increases their sensitivity to temozolomide by disrupting the NONO–SFPQ complex
Source: Cell Death Dis. 2024 Aug 25;15(8):616. doi: 10.1038/s41419-024-07010-z (PMC11345445; doi:10.1038/s41419-024-07010-z)
Supplement: Supplementary file 1 — Supplementary materials [file 41419_2024_7010_MOESM1_ESM.docx]

**
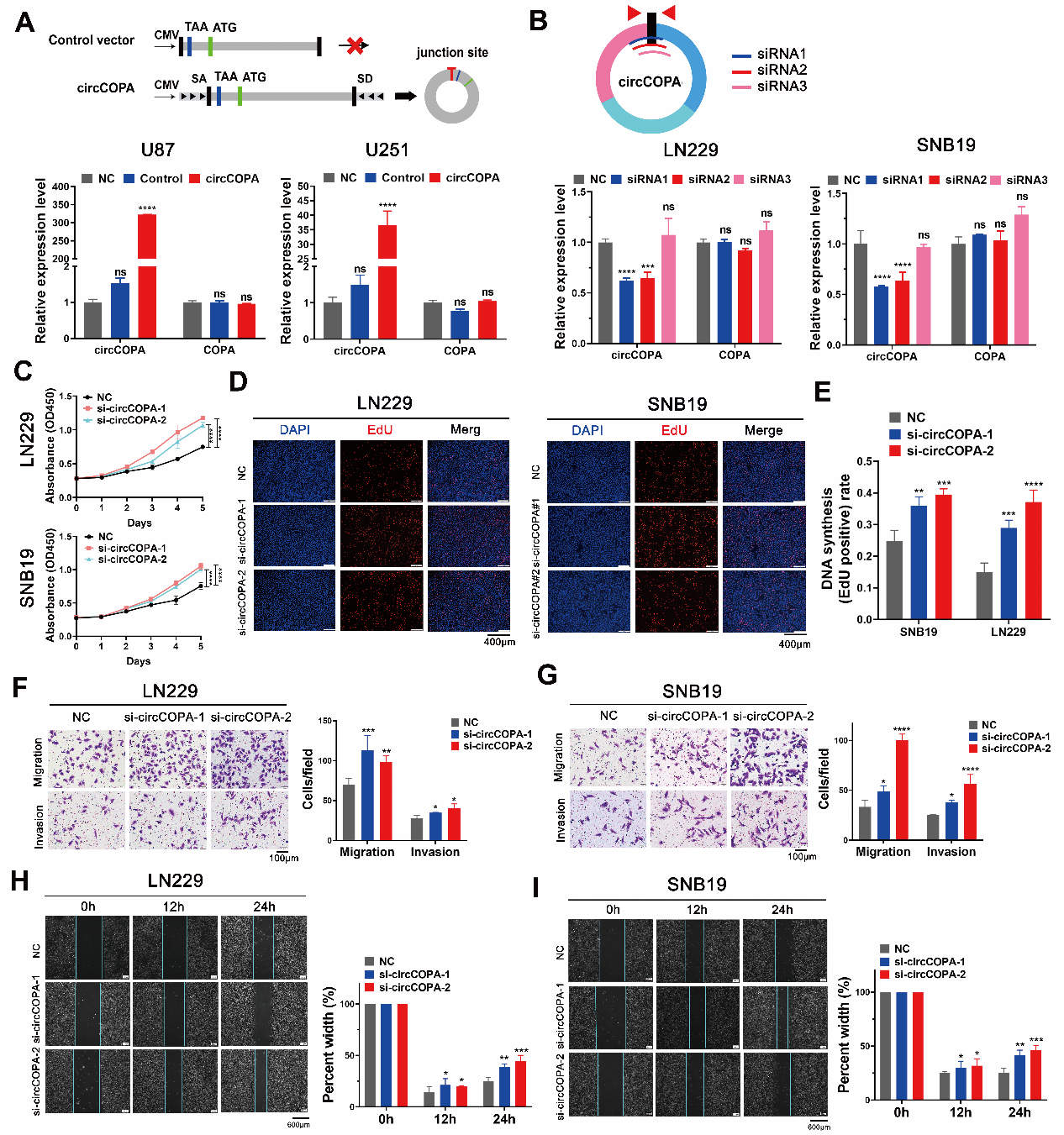
**

**Supplementary Figure 1 Tumor suppressive functions of** **circCOPA in GBM cell lines. (A)** Upper panel: illustration of the circCOPA overexpression plasmid and control plasmid. In the control vector, the flanking sequences were deleted, which prevents the formation of circCOPA. CircCOPA-overexpressing plasmid: a plasmid with intact flanking sequences, a splicing acceptor (SA) and a splicing donor (SD) on both sides of the circCOPA sequence. Lower panel: circCOPA expression was increased in U87 and U251 cells after transfection with the circCOPA overexpression plasmid, but COPA mRNA expression did not change (n = 3). **(B)** Upper panel: three siRNAs targeting the circCOPA junction site were used. Lower panel: The knockdown efficiency of siRNAs and their effect on parental genes were verified in LN229 and SNB19 cells (n = 3). CCK8 (**C**) and EdU (**D-E**) assays were used to detect the proliferation of LN229 and SNB19 cells after the inhibition of circCOPA (n = 3). **D**, Scale bar = 400 μm. Transwell (**F-G**) and wound healing (**H-I**) assays verified that circCOPA silencing promoted the migration and invasion of LN229 and SNB19 cells (n = 3). **F**, **G**, Scale bar = 100 μm. **H, I** Scale bar = 600 μm. All data were shown as mean ± SD (bar plots). **p* < 0.05, ***p* < 0.01, ****p* < 0.001, *****p* < 0.0001, unpaired two-tailed Student’s t test.


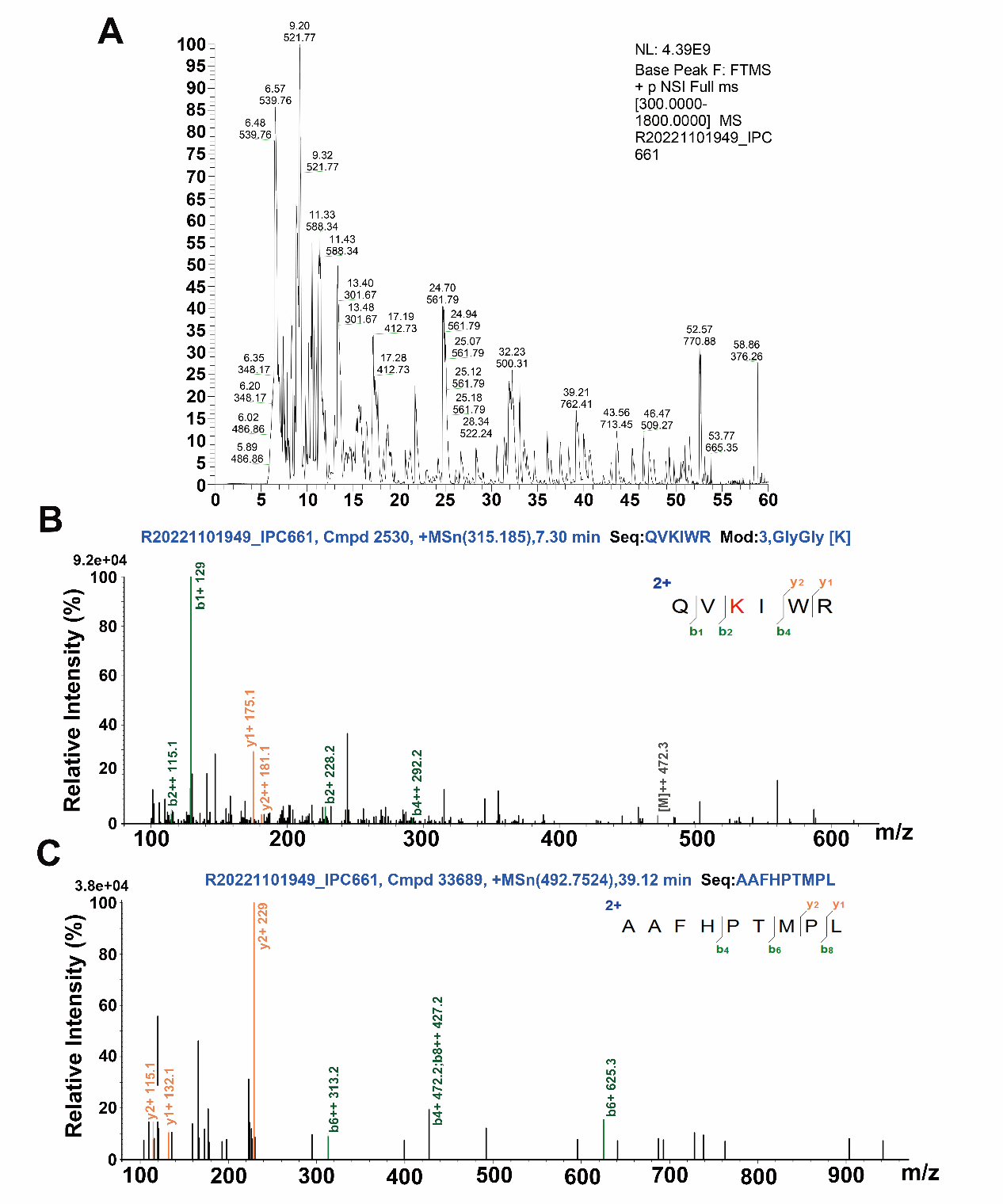


**Supplementary Figure 2 LC–MS analysis of COPA-99aa.** **(A)** LC‒MS/MS analysis of the total proteins. **(B-C)** LC–MS/MS-identified amino acid sequences of COPA-99aa (QVKIWR and AAFHPTMPL).

**
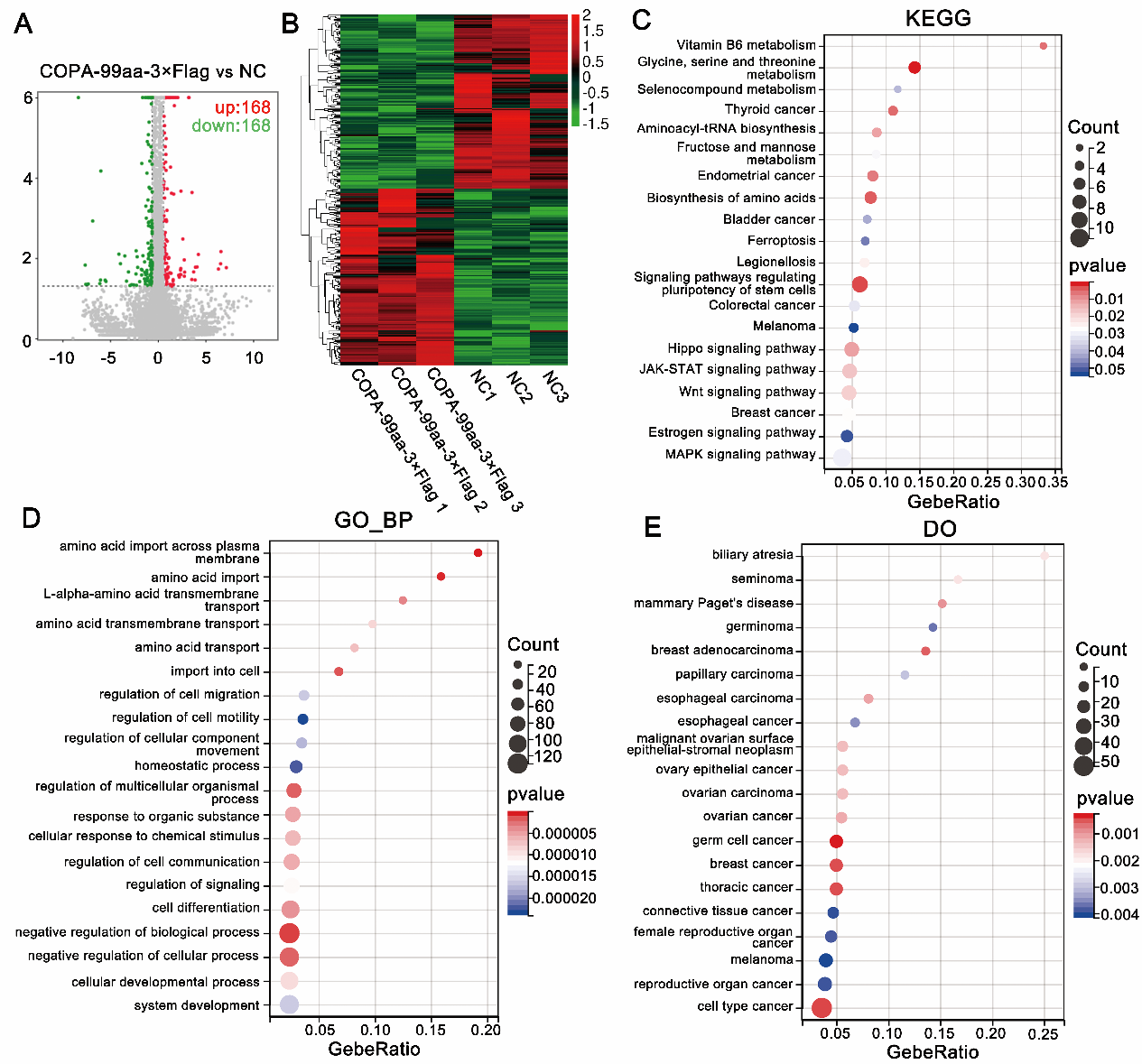
**

**Supplementary Figure 3. RNA-seq analysis of U87 cells overexpressing circCOPA and the corresponding control cells.** Volcano map **(A)** and heatmap **(B)** showed that 236 differentially expressed genes (DEGs) were detected (168 downregulated and 168 upregulated) in U87 cells overexpressing circCOPA. KEGG (**C**), GO_BP (**D**) and DO (**E**) analyses of DEGs.

**
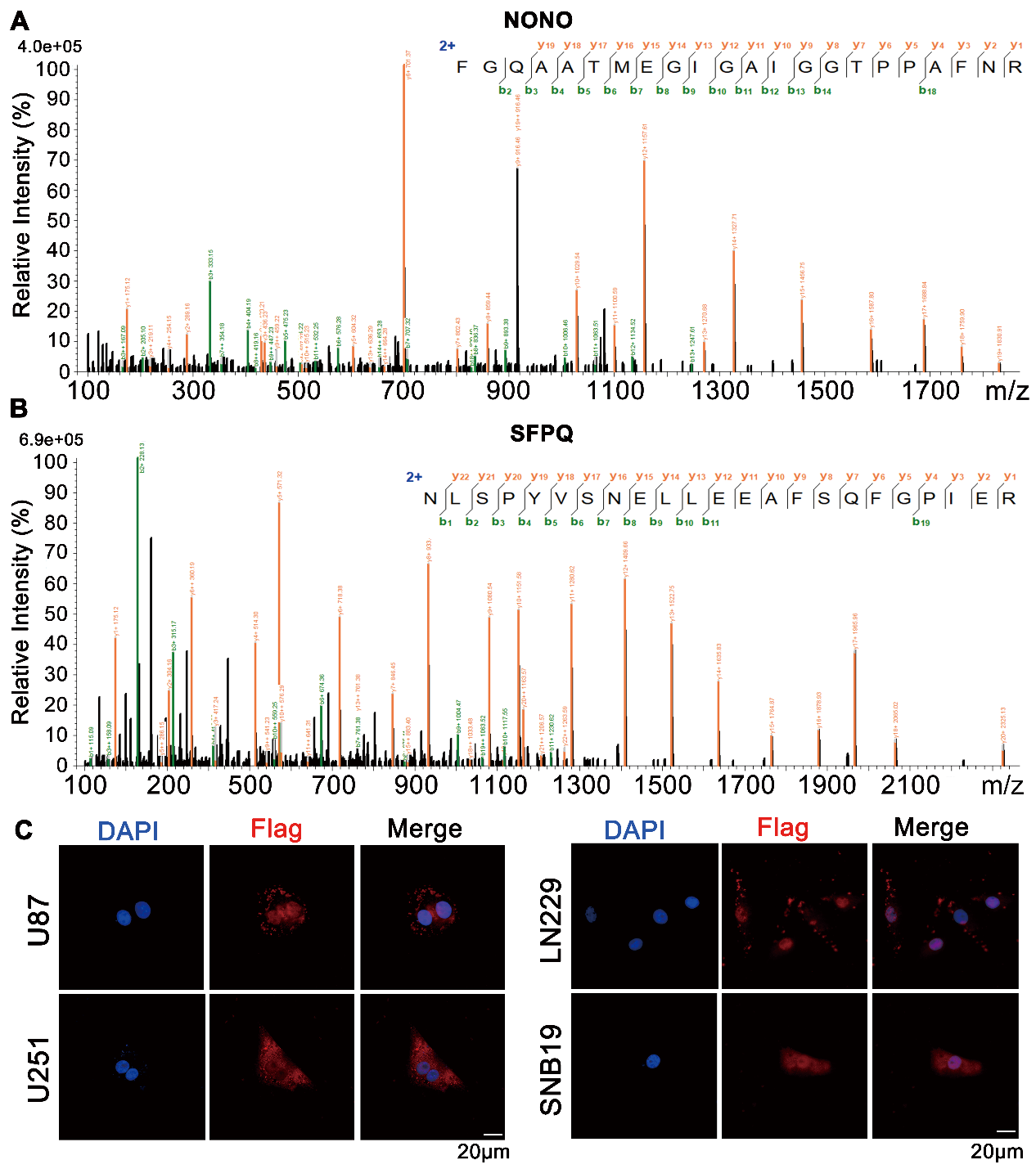
**

**Supplementary Figure 4 Analysis of COPA-99aa binding to the NONO and SFPQ** **proteins. (A-B)** LC–MS analysis of COPA-99aa-binding proteins (NONO and SFPQ). **(C)** COPA-99aa was both localized in the cytoplasm and nucleus. Scale bar = 20 μm.


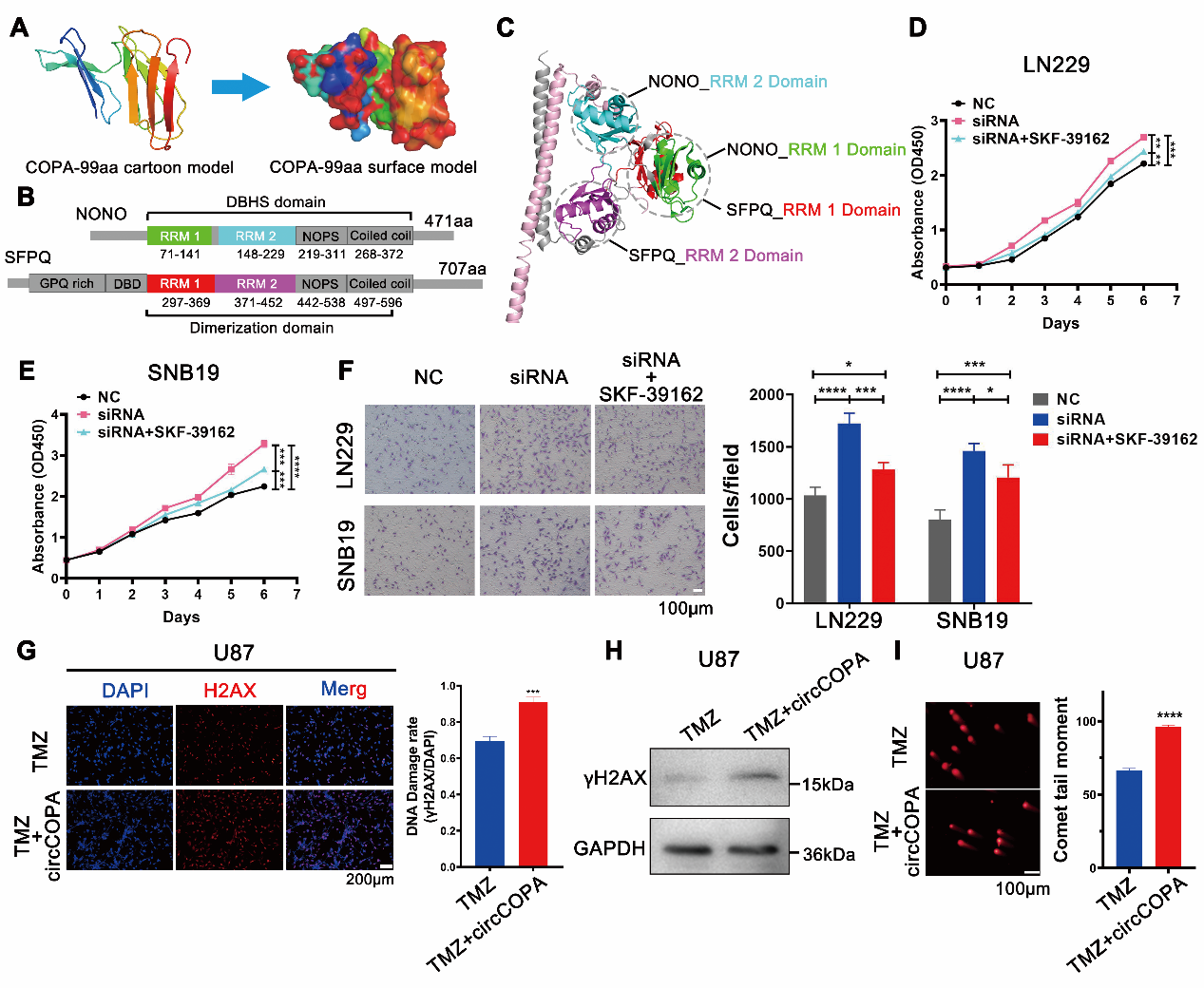


**Supplementary Figure 5 CircCOPA stimulated the DNA damage caused by TMZ in GBM cells**

**(A)** The 3D structure of COPA-99aa (cartoon and surface model) was predicted via the I-TASSER server. **(B)** Schematic representation of the NONO and SFPQ protein domain architectures. **(C)** Visualization of the 3D structure of the NONO and SFPQ dimers by using PyMol (PDB ID: 6WMZ). **(D-E)** knockdown of circCOPA promoted the proliferation of LN229 and SNB19 cells, but simultaneous disruption of the NONO–SFPQ complex partly reversed their growth (n = 3). **(F)** CircCOPA silencing stimulated the invasion of LN229 and SNB19 cells, but the increase of invasion was partly eliminated after simultaneous disruption of the NONO–SFPQ complex (n = 3). Scale bar = 100 μm. **(G-H)** CircCOPA overexpression promoted the expression of γH2AX caused by TMZ treatment in U87 cells (n = 3). Scale bar = 200 μm. (**I**) Comet assays showed circCOPA overexpression promoted the DNA damage induced by TMZ in U87 cells (n = 3). Scale bar = 100 μm. All data were shown as mean ± SD (bar plots). **p* < 0.05, ***p* < 0.01, ****p* < 0.001, *****p* < 0.0001, unpaired two-tailed Student’s t test.
