# Supplementary material for: A novel protein encoded by circCOPA inhibits the malignant phenotype of glioblastoma cells and increases their sensitivity to temozolomide by disrupting the NONO–SFPQ complex
Source: Cell Death Dis. 2024 Aug 25;15(8):616. doi: 10.1038/s41419-024-07010-z (PMC11345445; doi:10.1038/s41419-024-07010-z)
Supplement: Supplementary file 2 — Supplementary Table 1 [file 41419_2024_7010_MOESM2_ESM.docx]

**Supplementary Table 1. Clinical data of 36 GBM patients.**

| Case ID | Age | Gender | Grade | OS（months） | censor Yes=1; No=0 | circCOPA expression |
| --- | --- | --- | --- | --- | --- | --- |
| 1 | 49 | Male | Grade 4 | 7.8 | 1 | 18.75849 |
| 2 | 70 | Male | Grade 4 | 12.4 | 1 | 12.40884 |
| 3 | 54 | Male | Grade 4 | 49.53 | 0 | 11.41539 |
| 4 | 46 | Male | Grade 4 | 10.3 | 1 | 9.953104 |
| 5 | 27 | Famale | Grade 4 | 13.2 | 0 | 9.292425 |
| 6 | 37 | Male | Grade 4 | 46.37 | 0 | 6.133845 |
| 7 | 60 | Famale | Grade 4 | 12.5 | 1 | 5.185111 |
| 8 | 71 | Famale | Grade 4 | 8.2 | 1 | 4.368391 |
| 9 | 64 | Male | Grade 4 | 10.2 | 1 | 4.289787 |
| 10 | 27 | Male | Grade 4 | 25.6 | 1 | 2.9502 |
| 11 | 57 | Male | Grade 4 | 22.67 | 1 | 2.79493 |
| 12 | 51 | Male | Grade 4 | 41.3 | 1 | 2.521736 |
| 13 | 62 | Famale | Grade 4 | 42.4 | 1 | 1.956421 |
| 14 | 46 | Male | Grade 4 | 47.04 | 0 | 1.921673 |
| 15 | 58 | Male | Grade 4 | 16.4 | 1 | 1.881414 |
| 16 | 47 | Male | Grade 4 | 43.4 | 0 | 1.859978 |
| 17 | 57 | Male | Grade 4 | 10.1 | 1 | 1.855323 |
| 18 | 68 | Famale | Grade 4 | 25.43 | 1 | 1.602862 |
| 19 | 49 | Male | Grade 4 | 24 | 1 | 1.452973 |
| 20 | 51 | Famale | Grade 4 | 19.3 | 1 | 1.395356 |
| 21 | 52 | Famale | Grade 4 | 24.6 | 1 | 1.138973 |
| 22 | 63 | Famale | Grade 4 | 19 | 1 | 1.13565 |
| 23 | 74 | Male | Grade 4 | 10.8 | 1 | 1.049032 |
| 24 | 40 | Famale | Grade 4 | 17.47 | 0 | 0.955612 |
| 25 | 62 | Famale | Grade 4 | 21.7 | 1 | 0.953499 |
| 26 | 56 | Famale | Grade 4 | 0.9 | 1 | 0.852775 |
| 27 | 62 | Male | Grade 4 | 4.8 | 1 | 0.844696 |
| 28 | 28 | Famale | Grade 4 | 11.2 | 1 | 0.813604 |
| 29 | 53 | Male | Grade 4 | 10.5 | 1 | 0.750846 |
| 30 | 57 | Male | Grade 4 | 10.9 | 1 | 0.720797 |
| 31 | 57 | Male | Grade 4 | 17.5 | 0 | 0.628667 |
| 32 | 69 | Famale | Grade 4 | 38.38 | 1 | 0.611783 |
| 33 | 60 | Famale | Grade 4 | 19.53333 | 1 | 0.407788 |
| 34 | 70 | Male | Grade 4 | 6.5 | 1 | 0.368948 |
| 35 | 68 | Male | Grade 4 | 11.9 | 1 | 0.229451 |
| 36 | 52 | Famale | Grade 4 | 24.9 | 1 | 0.153609 |
