# Supplementary material for: A novel protein encoded by circCOPA inhibits the malignant phenotype of glioblastoma cells and increases their sensitivity to temozolomide by disrupting the NONO–SFPQ complex
Source: Cell Death Dis. 2024 Aug 25;15(8):616. doi: 10.1038/s41419-024-07010-z (PMC11345445; doi:10.1038/s41419-024-07010-z)
Supplement: Supplementary file 3 — Supplementary Table 2 [file 41419_2024_7010_MOESM3_ESM.docx]

**Supplementary Table 2. RNA oligos and primers.**

| **siRNAs** | **sense 5'-3'** | **antisense 5'-3'** |
| --- | --- | --- |
| siRNA1 | UCUGGCGCAUGAAUGUGUGTT | CACACAUUCAUGCGCCAGATT |
| siRNA2 | CUGGCGCAUGAAUGUGUGUTT | ACACACAUUCAUGCGCCAGTT |
| siRNA3 | UGGCGCAUGAAUGUGUGUUTT | AACACACAUUCAUGCGCCATT |
| **Primers** | **Forward primer 5'-3'** | **Reverse primer 5'-3'** |
| GAPDH | GGTGGTCTCCTCTGACTTCAACA | GTTGCTGTAGCCAAATTCGTTGT |
| COPA | TATGCTGGCACAGGCAATCT | ATCACAATGGCGTGTTTGGC |
| circCOPA | CTGGCGCATGAATGTGTGTT | CTTCTGTGGGGTGGAACTGAG |
| U6 | CTCGCTTCGGCAGCACA | AACGCTTCACGAATTTGCGT |
| b-actin | ACAGAGCCTCGCCTTTGCCGAT | CTTGCACATGCCGGAGCCGTT |
| Divergent primer | ATCGTGGAGTAAACTGGGCTG | TCTTCTGTGGGGTGGAACTG |
| Convergent primer | CAGTTCCACCCCACAGAAGA | GAAATATCCCAAACGCGCACA |
| CDH1 | ATTTTTCCCTCGACACCCGAT | TCCCAGGCGTAGACCAAGA |
| CDH2 | AGCCAACCTTAACTGAGGAGT | GGCAAGTTGATTGGAGGGATG |
| SNAI1 | CTCGGACCTTCTCCCGAATG | AAAGTCCTGTGGGGCTGATG |
| CCN4 | AGGTCAGCCCGTTTCAGAAG | TAGAGGAGTGTTCCAGGGCA |
| ZEB1 | ATGATGAATGCGAGTCAGATGC | ACAGCAGTGTCTTGTTGTTGT |
| TWIST1 | CGGCCAGGTACATCGACTTC | CAGAGGTGTGAGGATGGTGC |
