# Supplementary figures and images for: A novel protein encoded by circCOPA inhibits the malignant phenotype of glioblastoma cells and increases their sensitivity to temozolomide by disrupting the NONO–SFPQ complex
Source: Cell Death Dis. 2024 Aug 25;15(8):616. doi: 10.1038/s41419-024-07010-z (PMC11345445; doi:10.1038/s41419-024-07010-z)

**Original western blots used in this study.**


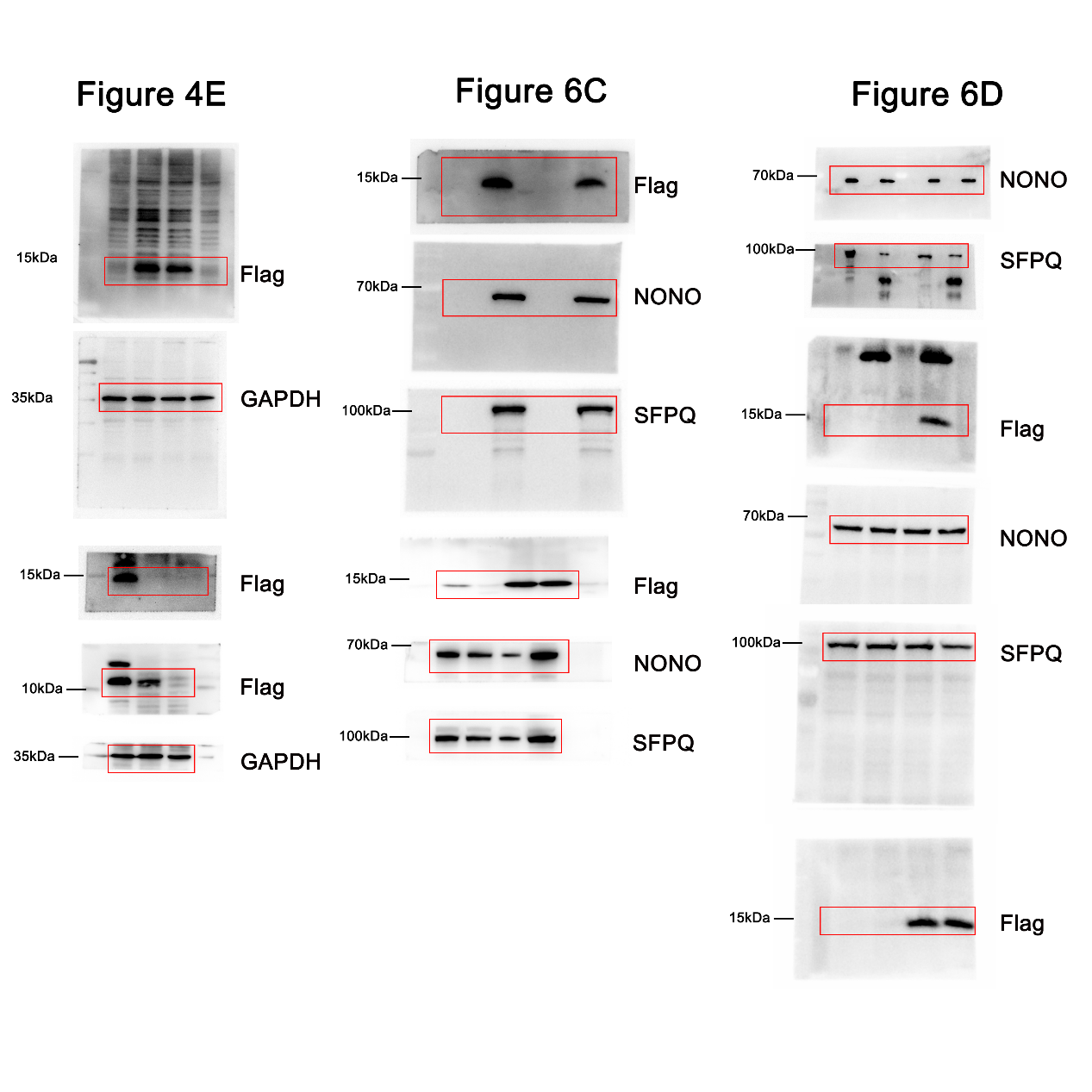

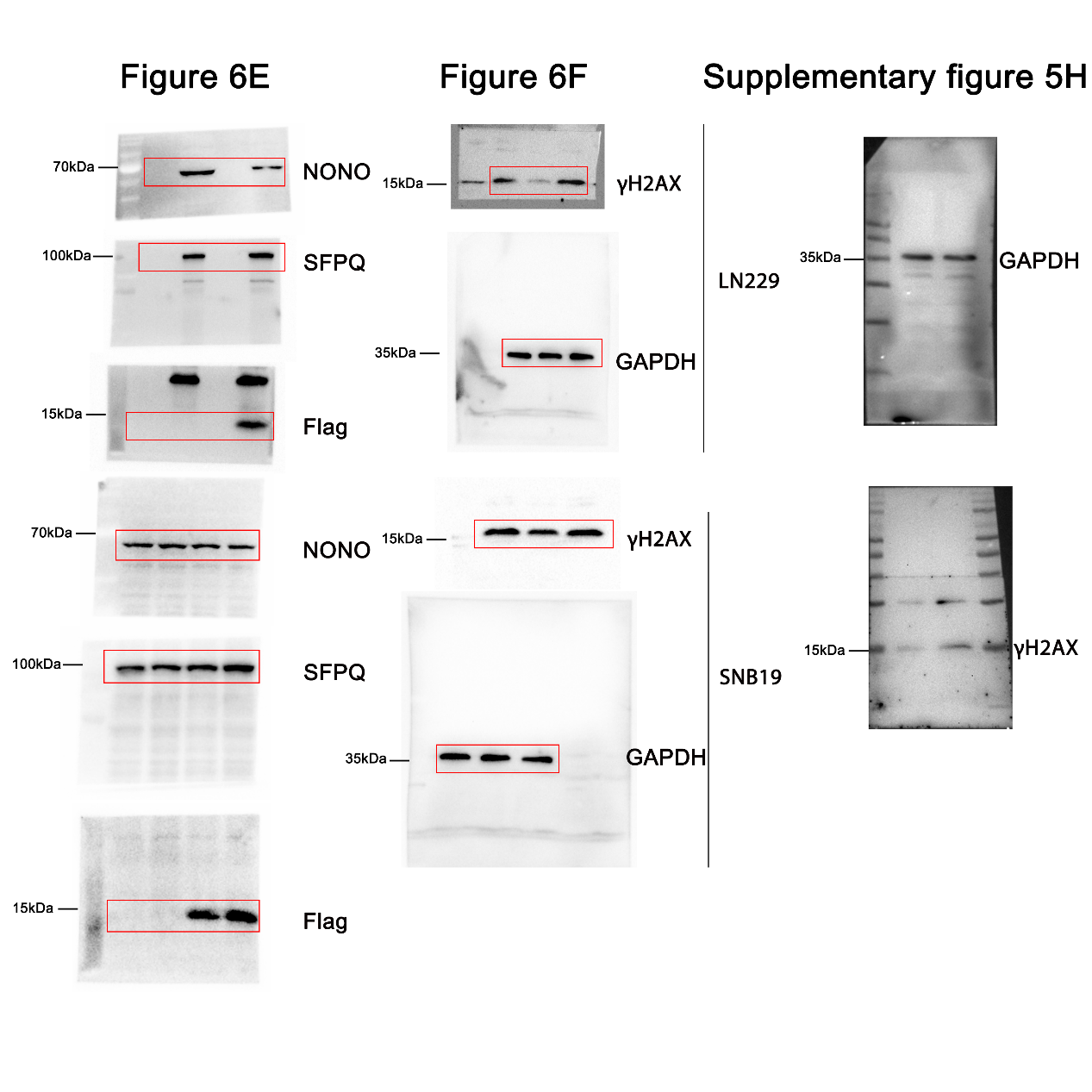

Supplement: Supplementary file 4 — Original western blots [file 41419_2024_7010_MOESM4_ESM.docx]
